# HPV vaccination policies and implementation for adolescents in low- and middle-income countries: a scoping review

**DOI:** 10.1016/j.lanwpc.2026.101919

**Published:** 2026-07-08

**Authors:** Shudan Liu, Di Wu, Siyan Ye, Yuqing Xiao, Meng Wu, Shu Chen, Xinyu Zhang, Lei Guo, Qin Zhang, Yang Zhou, Xiangju Wu, Qian Yang, Rongchang Xiao, Jie Zhang, Xinyi Feng, Jinghan Wang, Xianwen Shi, Xiaoyuan Zhang, Jiawei Xu, Jianchao Shao, Shenglan Tang, Qin Liu

**Affiliations:** a Research Center for Environment and Human Health, Research Center for Medicine and Social Development, School of Public Health, Chongqing Medical University, Chongqing, China; b School of Nursing, Chongqing Medical University, Chongqing, China; c National Centre for Immunisation Research and Surveillance, New South Wales, Australia; d Duke Global Health Institute, Duke University, NC, USA; e Global Health Research Center, Duke Kunshan University, Kunshan, Jiangsu, China; f Chongqing Center for Disease Control and Prevention (Chongqing Academy of Preventive Medicine), Chongqing, China; g Immunization Program Management Department, Chongqing Shapingba District Center for Disease Control and Prevention, Chongqing, China

**Keywords:** HPV vaccination, Adolescents, Low- and middle-income countries (LMICs), Vaccination policies, Implementation, Scoping review

## Abstract

This scoping review mapped adolescent HPV vaccination policies across 84 low- and middle-income countries (LMICs) with national HPV immunization programs and synthesized implementation evidence through July 2025. Focusing on adolescents aged 9–14 years, we identified policy convergence in target age, school-based delivery, and free vaccination provision, alongside divergence in gender eligibility, vaccine type, and funding mechanisms across income levels. Nearly half of LMICs have adopted single-dose schedules, reflecting a clear trend toward dosing simplification. Despite these policy advances, only 9.8% of countries achieved the WHO 90% complete vaccination target among females by age 15. Coverage analysis suggested that school-based programs maintained more stable performance over time than health facility-based approaches. Implementation evidence identified four facilitating factors, while barriers varied by income level. School-based programs complemented by mixed delivery approaches and single-dose schedules may offer a promising pathway toward the 2030 cervical cancer elimination goals.

**Research in context**

**Evidence before this study**

We searched PubMed, Web of Science, Cochrane Library, and EMBASE of Evidence Synthesis for scoping and systematic reviews from database inception through July 31, 2025. Search terms combined three domains: (1) HPV vaccination, (2) strategies or implementation outcomes (e.g., “strategies”, “policies”, “implementation”, “experiences”, “barriers”, “hesitancy” and “equity”), and (3) Western Pacific country identifiers. Current literature often focuses on single aspects of HPV vaccination, lacking comprehensive synthesis of policy landscapes and implementation experiences across different LMIC contexts. This evidence gap hinders the optimization of vaccination strategies in resource-constrained settings.

**Added value of this study**

In this scoping review, we mapped HPV vaccination policies across 84 LMICs, tracking their evolution and synthesizing implementation evidence through July 2025. We found that only 9.8% of countries met the WHO 90% coverage target. We documented a clear evidence-based policy evolution from three-dose to single-dose schedules (now adopted by nearly half of LMICs), and suggest that school-based programs tend to maintain more stable coverage over time compared to health facility-based approaches. We also identified four critical success factors: robust policy design, effective service delivery, tailored communication, and sustained capacity building, while barriers varied by income level.

**Implications of all available evidence**

Our findings suggest that school-based vaccination combined with single-dose schedules may offer a most promising pathway for LMICs to achieve the 2030 cervical cancer elimination goals. It also suggests areas for future research, including comparative effectiveness research on school-based versus mixed-method approaches using implementation science methods, and evaluate the long-term effectiveness of single-dose vaccination to confirm public health impact.

## Introduction

Cervical cancer is the fourth most common cancer in women globally. According to WHO data, approximately 660,000 new cases and around 350,000 deaths from cervical cancer occurred worldwide in 2022.^1^ The burden of cervical cancer is disproportionately distributed, with low- and middle-income countries (LMICs) bearing over 85% of global cases and deaths.^2^^,^^3^ Nearly all cervical cancer cases are caused by human papillomavirus (HPV), a sexually transmitted infection.^4^ Without effective preventive interventions, cervical cancer cases and deaths are projected to continue increasing among all WHO regions, driven by population growth and aging.^3^

The development of HPV vaccines represents a transformative opportunity for cervical cancer prevention. Extensive evidence demonstrates that HPV vaccines are safe, effective, and provide long-lasting protection against oncogenic HPV types responsible for most cervical cancers.^5^^,^^6^ In 2020, the World Health Assembly adopted the *Global Strategy to Accelerate the Elimination of Cervical Cancer*, setting a target of reducing cervical cancer incidence below 4 per 100,000 women, with 90% of girls fully vaccinated by age 15 by 2030.^7^

Despite widespread recognition of HPV vaccination effectiveness, gaps persist between LMICs and high-income countries. Barriers impede implementation in resource-constrained settings, including limited funding, weak health infrastructure, cultural barriers to vaccine acceptance, and insufficient political commitment amid competing health priorities.^8^ These challenges necessitate strategic priority-setting that optimizes resource allocation and maximizes population health impact.

Targeting adolescents aged 9–14 years for HPV vaccination offers distinct advantages. Immunologically, vaccination prior to sexual debut ensures maximum protection,^9^ and adolescents in this age group demonstrate superior immune responses compared to older vaccinees, supporting simplified dosing schedules.^10^^,^^11^ Programmatically, school-based delivery platforms capitalize on high enrollment rates in many LMICs, providing cost-efficient access to the target populations.^12^

Despite these advantages, research gaps remain. LMICs have adopted diverse vaccination policies regarding rollout models, service delivery approaches, dose schedule and other aspects. However, the effectiveness of these vaccination policies across different contexts have not been sufficiently synthesized. Moreover, evidence on implementation challenges and experiences remains inadequately evaluated. Current literature often focuses only on a single aspect of HPV vaccination policies or implementation outcomes, failing to comprehensively depict the complete process from strategy design to actual execution.

Given the breadth of this topic, this study adopts a scoping review methodology, aiming to: (1) systematically map HPV vaccination policies across LMICs, including the current policy frameworks and their evolution over the years, and the vaccination coverage under different vaccination strategies; (2) synthesize evidence on the implementation experiences and barriers. This study will provide evidence to help LMICs in developing and optimizing adolescent HPV vaccination strategies under resource constraints.

## Methods

This scoping review used a two-step approach to examine HPV vaccination policies and implementation in LMICs, focusing on adolescents aged 9–14 years. Step 1 identified national HPV vaccination policies, including introduction year, rollout mode, target population, dose schedule, vaccine type, delivery platform, funding sources, cooperation mechanisms, and reported coverage. Step 2 synthesized evidence on implementation, highlighting experiences, barriers, vaccine hesitancy, and equity concerns. We followed the PRISMA extension for Scoping Reviews^13^ to guide result presentation (Supplemental Table S1).

### Information sources and search strategy

For policies identification, we conducted a stepwise search of the following sources: 1) WHO vaccine dashboard and official health ministry websites of each country; 2) websites of international organizations including Gavi (The Global Alliance for Vaccines and Immunization), PATH (Program for Appropriate Technology in Health), and CHAI (Clinton Health Access Initiative); 3) electronic databases including PubMed and Web of Science. We also prepared a standard email to relevant policy makers, project officials and researchers as supplementary searches. No language restrictions were applied. Search terms included “HPV vaccination” OR “HPV immunization” OR “cervical cancer prevention”. Once a program was identified through searches of official government and public health agency websites, additional keywords were manually searched to extract information on the introduction year, rollout/delivery mode, target population, vaccination/dose schedule, vaccine type, primary delivery strategy, funding, collaboration, and coverage. When multiple versions of a policy document were available, only the most recent version in effect at the time of data extraction was included. All website searches were completed by July 31, 2025. No language restrictions were applied. Data on introduction year, rollout mode, and vaccination coverage were obtained from the WHO website.

For implementation evaluation, we searched PubMed, Web of Science, Cochrane Library, and EMBASE from database inception through July 31, 2025 (supplementary Table S2). The search terms combined “HPV vaccination” or “cervical cancer prevention” with “implementation”, “experiences”, “barriers”, “hesitancy” and “equity”. No language restrictions were applied in the search. Search results were exported to EndNote ×9 for duplicate removal.

### Eligibility criteria

Our review focused on 84 LMICs that incorporated HPV vaccination into their national immunization programs (NIP), according to World Bank Fiscal Year 2026 classifications. The population comprised adolescents eligible for HPV vaccination. For policy identification, eligible sources included government documents, policy reports, official guidelines, and articles describing HPV vaccination programs, policies, strategies, recommendations, or implementation approaches. Documents were excluded if they did not address HPV vaccination policies or programs, or if sufficient policy information could not be extracted. For implementation evaluation, eligible sources included reports and peer-reviewed quantitative, qualitative, and mixed-methods studies examining issues about implementation experiences and barriers, hesitancy, and equity. Studies conducted in countries not included in Step 1 or lacking implementation-related information were excluded. When multiple studies from the same country were identified, data were extracted from all eligible studies and integrated into the synthesis. The primary citation was then selected according to study design: for quantitative studies, the most recently published study was prioritized to best reflect current programme status; for qualitative studies, the study providing the most nationally representative evidence was selected to minimize local or subnational reporting bias.

### Screening

Two reviewers independently retrieved all records from databases in two stages: title and abstract screening followed by full text review. Screening was conducted using Endnote ×9. Disagreements were resolved through discussion, with a third reviewer (QL) consulted if needed. Non-English documents were translated using Microsoft Edge/Google Chrome Translator. To minimize translation errors, key information was independently reviewed by two investigators and cross-checked against the original source documents when necessary.

### Data extraction and synthesis

We used standardized data extraction forms for policy identification and implementation evaluation. Items were defined based on review objectives, existing literature, and team discussion. For policy identification, extracted variables included introduction year, rollout mode, target population, dose schedule, vaccine type, delivery platform, funding sources, coordination mechanisms and coverage by age 15. For each of these variables, information documented in at least one source was sufficient for it to be recorded as present.

For implementation evaluation, we extracted data on experiences, barriers, hesitancy, and equity. Data were synthesized using descriptive and thematic approaches. Descriptive data were organized in tables summarizing strategies and implementation features by country. Thematic synthesis identified key patterns related to implementation experiences, barriers, hesitancy, and equity. The thematic synthesis followed a hybrid deductive-inductive approach.^14^^,^^15^ An analytical framework comprising four domains for implementation experiences (policy and institutional design; service delivery and collaboration; communication strategies; capacity building) and four domains for barriers (system, sociocultural, knowledge, and resource barriers) was developed a priori through team discussion and informed by existing implementation science literature, constituting the deductive component of the synthesis. Within each predefined domain, coding was applied inductively to capture country-specific patterns not anticipated by the framework. Two reviewers (SDL and DW) independently coded all extracted data using this framework, with discrepancies resolved through discussion. Where consensus could not be reached, a senior reviewer (QL) adjudicated. Themes were iteratively refined through two rounds of discussion among the core research team to ensure analytical consistency.

For coverage analysis, national HPV vaccination coverage data for four indicators (15HPV1_F, 15HPVC_F, 15HPV1_M, 15HPVC_M) were retrieved from the WHO immunization dashboard. Country-level 2024 coverage values were plotted as scatter plots to characterize the distribution of current programme performance. To examine temporal trends by vaccination policies (delivery platform, dose regimen and rollout mode), unweighted arithmetic means of country-specific annual coverage values were calculated within each strategy group for each calendar year, with bubble size used to represent the number of contributing countries per group-year estimate.

## Results

For policy identification, strategies from 84 included countries were identified through the following websites: WHO (n = 55), official health ministry websites (n = 54), Gavi (n = 16), IVAC (n = 10), UICC (n = 4), UNFPA (n = 2), UNICEF (n = 1) and official news websites (n = 7). For certain countries where specific policies could not be located through these websites, literature was searched and extracted from the PubMed (n = 34) and Web of Science (n = 1) databases.

For implementation evaluation, the search identified 8483 records through database searches, including PubMed (n = 3241), Web of Science (n = 2606), Embase (n = 2413), and the Cochrane Library (n = 223), together with four studies included in the previous version of the review. After removal of 3136 duplicate records, 5347 records were screened by title and abstract, of which 5006 were excluded. Full texts were sought for 341 records, and 60 could not be retrieved. The remaining 281 full text articles were assessed for eligibility, leading to the exclusion of 260 records due to mismatched study type, study area, intervention, outcomes, participants, pooled multi country data, or lack of national representativeness. Ultimately, 20 studies met the inclusion criteria and were included in the review, resulting in a total of 24 studies included in the final synthesis, as the process shown in Supplemental Figure S1.

### HPV vaccination policies in LMICs

**Fig. 1:**
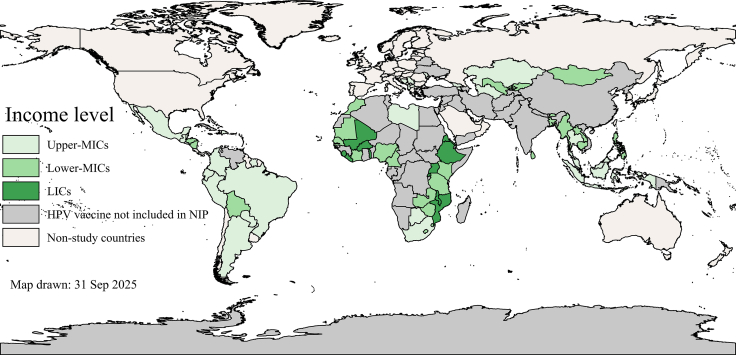
**Countries included in the study**. Notes: Upper-MICs, upper-middle-income countries; lower-MICs, lower-middle-income countries; LICs, low-income countries; HPV, human papillomavirus; NIP, national immunization programs.

Among 84 LMICs, 39 upper-middle-income countries (upper-MICs), 33 lower-middle-income countries (lower-MICs) and 12 low-income countries (LICs) included HPV in their NIPs (Fig. 1). We documented both current vaccination policies (Table 1) and their temporal evolution (Supplemental Table S3).

**Table 1:** Characteristics of adolescent HPV vaccination strategies in 84 LMICs.

Income level	Country (region)	Introduction year	Last update year	Age (year)/target population	Sex	Doses	Vaccine type	Delivery platform	Payment strategy	Funding source
Upper-MICs	Albania	2022	NA	13	F	1 dose	Gardasil (4v)	Health facility-based	free	GF and EF
	Argentina	2011	2024	11–20	F and M	1 dose	Gardasil (4v)	Health facility-based	free	GF
				11–26 ICP	F and M	3 doses (0, 2, 6 m)	Gardasil (4v)			
	Armenia	2017	2019	13–45	F	2 doses (0, 6 m)	Gardasil (4v)	Health facility-based	free	GF and EF
				14–45	M	2 doses (0, 6 m)	Gardasil (4v)			
	Belize	2016	2023	9–15	F and M	1 dose	Gardasil (4v)	School- based/health facilities (unschooled)	free	GF
	Bosnia and Herzegovina (FBiH. Canton Sarajevo)	2022	NA	11–26	F	2 doses (0, 6 m)	Gardasil (4v)	Health facility-based	free	NA
	Bosnia and Herzegovina (FBiH. Elsewhere in FBiH)	2023	NA	13–14	F	2 doses (0, 6 m)	Gardasil (4v)	Health facility-based	free	NA
	Bosnia and Herzegovina (RS)	2023	NA	11–14	F and M	2 doses (0, 6 m)	Gardasil 9 (9v)	Health facility-based	free	NA
	Botswana	2015	2016	9 (Grade 5 in primary school)	F	2 doses (0, 6 m)	Gardasil (4v)	School-based/health facilities (unschooled)	free	NA
	Brazil	2014	2024	9–14	F and M	1 dose	Gardasil (4v)	Health facility-based	free	GF
				9–45 ICP	F and M	3 doses (0, 2, 6 m)	Gardasil (4v)			
	Colombia	2012	2024	9–17	F and M	1 dose	Gardasil (4v)	School-based	free	GF
	Dominica	2019	2023	10–12	F and M	1 dose	Gardasil (4v)	School-based	free	GF
	Dominican Republic	2017	NA	9–14	F	1 dose	Gardasil (4v)	Health facility-based	free	NA
	Ecuador	2014	NA	9–11	F	2 doses (0, 6 m)	Cervarix (2v)	School-based	free	NA
	El Salvador	2020	NA	9	F	2 doses (0, 6 m)	Gardasil (4v)	School-based	free	GF and EF
	Fiji	2013	2015	8–13	F	2 doses (0, 6 m)	Cervarix (2v)	School-based	free	GF and EF
	Georgia	2019	2023	10–46 F and 10–26 M	F and M	2 doses (≤14 years, 0, 6 m) 3 doses (≥15 years, 0, 2, 6 m)	Gardasil (4v)	Health facility-based	free	GF and EF
	Grenada	2019	2023	9–10	F and M	1 dose	Gardasil (4v)	School-based	free	GF and EF
	Guatemala	2018	2024	9–17 F and 9 M	F and M	1 dose	Gardasil (4v)	School-based	free	NA
	Indonesia	2017	NA	11	F	2 doses (0, 12 m)	Gardasil (4v)	School-based	free	GF and EF
	Jamaica	2017	NA	9–14	F and M	1 dose	NA	School-based	free	NA
				15–26	F	2 doses (0, 6 m)				
				ICP	F and M	3 doses (0, 2, 6 m)				
	Kazakhstan	2024	2024	11	F	2 doses (0, 6 m)	Gardasil (4v)	NA	free	NA
	Libya	2013	2017	12	F	3 doses (0, 1, 6 m)	Gardasil (4v)	NA	free	GF and EF
	Malaysia	2010	2017	13	F	2 doses (0, 6 m)	Gardasil 9 (9v)	School-based	free	GF
	Maldives	2019	NA	10–14	F	2 doses (0, 6 m)	NA	School-based	free	GF and EF
	Marshall Islands	2009	NA	11–12	F	2 doses (0, 6 m)	NA	School-based	free	GF
	Mauritius	2016	2023	9–15	F and M	2 doses (0, 6 m)	Cervarix (2v)	School-based	free	GF
	Mexico	2012	2023	11–13	F	1 dose	Gardasil (4v)	School-based/health facilities (unschooled)	free	GF
				11–49 (with HIV)	F	3 doses (0, 1, 6 m)	Gardasil (4v)			
	Montenegro	2022	2023	9–14	F	1 dose	Gardasil 9 (9v)	Health facility-based	free	NA
	North Macedonia	2009	2024	12–19	F and M	3 doses	Gardasil 9 (9v)	School-based	free	GF
	Paraguay	2013	2024	9–14	F and M	1 dose	Gardasil (4v)	School-based	free	GF
	Peru	2015	2024	9–18	F and M	1 dose	Gardasil (4v)	School-based	free	GF and EF
	Republic of Moldova	2017	2021	9–14	F and M	2 doses (0, 6 m)	Gardasil (4v)	Health facility-based	free	GF
	Saint Lucia	2019	2025	11–12	F and M	1 dose	Gardasil 9 (9v)	School-based	free	GF
	Saint Vincent and the Grenadines	2017	NA	11–12	F	NA	Cervarix (2v)	School-based	free	GF and EF
	Serbia	2022	NA	9–14	F and M	2 doses (0, 6 m)	Gardasil 9 (9v)	Health facility-based	free	SHI
				15–19	F and M	3 doses (0, 2, 6 m)	Gardasil 9 (9v)			
	South Africa	2014	2024	≥9 in Grade 4 in primary school	F	1 dose	Cervarix (2v)	School-based	free	GF
	Suriname	2013	2023	9–13	F and M	2 doses (0, 6 m)	Gardasil (4v)	School-based	NA	NA
	Thailand	2017	NA	11 (Grade 5 in primary school)	F	2 doses (0, 6 m)	Gardasil (4v)	School-based	free	NA
	Tonga	2022	2023	10	F	1 dose	Cervarix (2v)	School-based/health facilities	free	NA
	Turkmenistan	2016	NA	9	F and M	2 doses (0, 6 m)	Gardasil (4v)	Mixed (schools and health facilities)	free	GF and EF
	Tuvalu	2021	NA	10	F	1 dose	Cervarix (2v)	NA	free	NA
Lower-MICs	Bangladesh	2023	NA	10–14	F	1 dose	Cervarix (2v)	Mixed (schools and health facilities for unschooled)	free	EF
	Bhutan	2010	2021	12	F and M	2 doses (0, 6 m)	Gardasil (4v)	School-based	free	GF
	Bolivia (Plurinational State of)	2017	2024	10	F and M	1 dose	Gardasil (4v)	School-based/health facilities (unschooled)	free	EF
	Cabo Verde	2021	2023	10	F	1 dose	Cervarix (2v)	School-based	NA	EF
	Cambodia	2023	NA	9	F	1 dose	Cervarix (2v)	School-based	free	GF and EF
	Cameroon	2020	2023	9	F and M	1 dose	Gardasil (4v)	Mixed (schools and health facilities)	free	EF
	Côte d'Ivoire	2019	2023	9	F	1 dose	Gardasil (4v)	School-based	free	GF and EF
	Eswatini	2023	NA	9–14	F	2 doses (0, 6 m)	Gardasil (4v)	School-based	free	GF
	Honduras	2016	2024	11	F	1 dose	Gardasil (4v)	School-based	free	EF
	Kenya	2019	NA	10–14	F	2 doses (0, 6 m)	Gardasil (4v)	Health facility-based	free	GF and EF
	Kiribati	2023	NA	9	F	1 dose	Gardasil (4v)	NA	NA	NA
	Kyrgyzstan	2022	NA	9–14	F	2 doses (0, 6 m)	Gardasil (4v)	School-based	NA	EF
	Lao People's Democratic Republic	2020	NA	10–14	F	2 doses (0, 12 m)	Gardasil (4v)	School-based	free	GF and EF
	Lesotho	2022	NA	9–14	F	2 doses (0, 6 m)	NA	School-based	free	EF
	Mauritania	2021	NA	9	F	2 doses (0, 6 m)	Gardasil (4v)	NA	NA	NA
	Micronesia (Federated States of)	2010	NA	10–11	F	2 doses (0, 6 m)	Gardasil 9 (9v)	School-based	NA	NA
	mongolia	2024	NA	11	F and M	1 dose	Gardasil (4v)	School-based	free	GF and EF
	Morocco	2022	NA	11	F	2 doses (0, 6 m)	Gardasil (4v)	School-based	free	GF and EF
	Myanmar	2020	NA	9–10	F	2 doses (0,12 m)	Gardasil (4v)	School-based	free	GF and EF
	Nicaragua	2023	NA	10–14	F	2 doses (0, 6 m)	Cecolin (2v)	Mixed (schools and health fairs, door-to-door visits, health facilities)	NA	NA
	Nigeria	2023	NA	9–14	F	1 dose	Gardasil (4v)	Mixed (schools and community centers)	free	GF and EF
	Philippines	2016	NA	9 (Grade 4 in primary school)	F	2 doses (0, 6 m)	Gardasil (4v)	School-based	free	GF
	Samoa	2022	2023	13 (Grade 8 in primary school)	F	1 dose	Cervarix (2v)	School-based	free	GF and EF
	Sao Tome and Principe	2021	2023	NA	F	1 dose	NA	NA	NA	NA
	Senegal	2018	NA	9	F	2 doses (0, 6–12 m)	Gardasil (4v)	School-based/health facilities	free	GF and EF
	Solomon Islands	2019	NA	9–14	F	2 doses	Gardasil 9 (9v)	School-based/community outreach strategy (unschooled)	NA	EF
	Sri Lanka	2017	NA	10–11	F	2 doses (0, 6 m)	Gardasil (4v)	School-based/health facilities (unschooled)	free	GF and EF
	Timor-leste	2024	2024	11–14	F	1 dose	Gardasil (4v)	School-based	free	GF and EF
	United Republic of Tanzania	2018	2024	9	F	1 dose	Gardasil (4v)	Mixed (schools, healthfacilities and community centers)	free	GF and EF
	Uzbekistan	2019	NA	9	F	2 doses (0, 6 m)	Gardasil (4v)	School-based	free	GF and EF
	Vanuatu	2023 (partial)	NA	9–13	F	1 dose	NA	Mixed (schools, health facilities and community centers)	free	GF and EF
	Zambia	2019	2023	9	F	1 dose	Gardasil (4v)	Mixed (schools, health facilities and community centers)	free	GF and EF
				9 (with HIV)	F	3 doses (0, 6, 12 m)				
	Zimbabwe	2018	2019	10 (Grade 5 in primary school)	F	2 doses (0, 12 m)	Cervarix (2v)	School-based/health facilities (unschooled)	free	GF and EF
LICs	Burkina Faso	2022	NA	9	F	1 dose	Gardasil (4v)	Mixed (schools and health facilities)	free	GF and EF
	Eritrea	2022	NA	9–14	F	2 doses (0, 6 m)	Gardasil (4v)	Mixed (schools and health facilities for remote villages)	free	EF
	Ethiopia	2018	2022	14	F	1 dose	Gardasil (4v)	Mixed (schools and health facilities for vulnerable communities)	free	EF
	Gambia	2019	2023	9–14	F	1 dose	Gardasil (4v)	Mixed (schools and community centers)	NA	EF
	Liberia	2019	NA	9–14	F	2 doses (0, 6 m)	Gardasil (4v)	Mixed (schools and health facilities)	NA	GF and EF
	Malawi	2019	NA	9	F	2 doses (0, 6 m)	Gardasil (4v)	Mixed (schools, health facilities and community centers)	free	GF and EF
	Mali	2024	2024	10	F	1 dose	Gardasil (4v)	Mixed (fixed, advanced, mobile facilities for vulnerable communities)	free	GF and EF
	Mozambique	2021	2022	9–14	F	2 doses (0, 6 m)	Cervarix (2v)	School-based/other strategy (door-to-door approach, mobile outreach, and vaccination at fixed facilities)	free	GF and EF
	Rwanda	2011	2015	12	F	2 doses (0, 6 m)	Gardasil (4v)	School-based	free	GF and EF
	Sierra Leone	2022	NA	10	F	2 doses	Cervarix (2v)	School-based	free	GF
	Togo	2023	NA	9–14	F	1 dose	Cervarix (2v)	School-based	free	EF and SHI
	Uganda	2015	NA	10	F	2 doses (0, 6 m)	Gardasil (4v)	School-based	free	GF and EF

Notes: Upper-MICs, upper-middle-income countries; Lower-MICs, lower-middle-income countries; LICs, low-income countries; ICP, Immunocompromised person; F, female; M, male; FBiH-CS, Federation of Bosnia and Herzegovina, Canton Sarajevo; FBiH-other, Federation of Bosnia and Herzegovina, Elsewhere; RS, Federation of Bosnia and Herzegovina, Republika Srpska; m, months; ys, years; GF, Goverment fund; EF, External fund; SHI, Social health insurance; NA, Not available.

### Timeline of HPV vaccine introduction in NIP

Upper-MICs led HPV introduction, with regional disparities in LMICs persisting. Among upper-MICs, Mexico pioneered regional vaccination programs in 2008, and was incorporated into the NIP in 2012; Bhutan was the first lower-MICs to pilot the program (2009), while Rwanda was the first LICs to introduce it (2011). Regionally, Latin America led in timing, with Africa and Asia relatively lagged behind. Six countries introduced HPV vaccination in 2024: Kazakhstan and Mongolia (upper-MICs), Bangladesh, Timor-Leste, and Vanuatu (lower-MICs), and Mali (LIC). Nine countries (Mexico, Malaysia, Brazil, Indonesia, Republic of Moldova, Philippines, Bangladesh, Nigeria, and Vanuatu) adopted phased rollout strategies, beginning with pilots before scaling up nationwide (Supplemental Table S4).

### Core policy features

We summarize design elements of adolescent HPV vaccination policies, including eligibility criteria, vaccine schedules and products, delivery platforms, financing arrangements, and coordination mechanisms.

#### Target age and gender

Most LMICs target ages 9–14, though vaccination of boys remains in upper-MICs. While lower-MICs and LICs predominantly focus on this age range, 11 upper-MICs have extended eligibility to broader age groups (up to 46). Among upper-MICs, 51.3% (20/39) also vaccinate boys, compared to only 4 lower-MICs and no LICs (S1 Table). Thirteen countries launched catch-up campaigns for age groups 1–7 years older than routine targets. During the evolution of policies over the years, 8 upper-MICs expanded target ages to wider ranges. Only 5 countries (6.0%) have formulated explicit vaccination strategies for immunocompromised populations (ICP), four of which are upper-MICs. Compared with the general population, these countries set a much wider eligible age range for ICP, extending up to 45–49 years (Supplemental Figure S2).

#### Doses and vaccine types

Dose schedules have progressively simplified from three-dose (2008–2013) to two-dose (2014–2022) to single-dose regimens (2023-present) (Supplemental Table S2). Currently, nearly half of LMICs have adopted single-dose schedules: 46.2% (18/39) of upper-MICs, 48% (16/33) of lower-MICs, and 41.6% (5/12) of LICs. Similar proportions maintain two-dose schedules, while three countries retain three-dose schedules. All the 5 countries maintain three-dose schedules for immunocompromised individuals (Supplemental Figure S3).

Quadrivalent vaccines dominate across income levels, used by 61.5% (24/39) of upper-MICs, 66.7% (22/33) of lower-MICs, and 75.0% (9/12) of LICs. Bivalent vaccines rank second. Since 2018, 6 upper-MICs and 2 lower-MICs have introduced the nonavalent vaccines, while no LIC has adopted them. Several countries adjusted their vaccine types during program implementation. For example, Argentina switched from the bivalent to the quadrivalent vaccine, while Bosnia and Herzegovina, Malaysia, North Macedonia, and Saint Lucia transitioned from the quadrivalent to the nonavalent vaccine (Supplemental Figure S4). The vast majority of countries using bivalent vaccines exclusively targeted girls, whereas quadrivalent and nonavalent vaccine adopters more frequently included boys.

#### Delivery platform

School-based delivery predominates: 66.7% (26/39) of upper-MICs, 66.7% (22/33) of lower-MICs, and 41.6% (5/12) of LICs. Mixed-method approaches rank second (1 upper-MIC, 7 lower-MICs, 7 LICs), while health facility-based delivery is least prevalent (10 upper-MICs, 1 lower-MIC) (Supplemental Figure S5 and Table 2).

**Table 2:** Summary characteristics of implementation experiences.

Income level	Country	Policy and institutional design	Service and collaboration	Communication strategy	Capacity building	Research method	Cite
Upper-MICs	Argentina	Strong leadership from the National Department of Health; Dedicated Organizations To Promote Diffusion; Adapt At A Provincial Level To Best Suit The Local Context	Health facility based	Address Concerns Of Health Care Professionals; Establish Communication Across Sectors And With The Public	Adequate Funding	Quali	^16^
	Brazil		School-based strategy		Electronic registry, Technology transfer	Quali	^17^
	Dominica Republic	Affordability policy	School-based strategy	Multi-channel publicity (hospitals, schools, churches)		Quali	^18^
	Peru		School-based strategy			Quali	^19^
	Serbia		School-based strategy	Pharmacy-based education (PBE) program (“Ask me about HPV”)		Quant	^20^
	South Africa	Careful planning and coordination and strong leadership from the National Department of Health	School-based strategy			Mixed	^21^
Lower-MICs	Bhutan	Affordability policy	School-based strategy	Multisectoral collaboration for social mobilization and advocacy	Digital monitoring and reporting systems	Quali	^22^
	Cambodia		School-based strategy	Standardized messaging	Teacher and Healthcare worker training	Quali	^23^
	Cameroon		Mixed (schools and health centers) method strategy	Sensitisationcampaigns conducted through local media using both radio and television		Quant	^24^
	Kenya		Health facility based strategy		Teachers as health promoters	Quali	^25^
	Nigeria		School-based strategy			Mixed	^26^
	Tanzania		Multi-sector collaboration	Social media strategy, Targeted messaging		Quali	^27^
	Zimbabwe		Health-Education cooperation, School campaign			Quali	^28^
LICs	Rwanda	Careful planning, coordination and political commitment	Internal and external partnerships			Quali	^29^
	Mali		Mixed (fixed, advanced, mobile facilities for vulnerable communities)	Community educational sessions by community health workers and a storytelling cloth		Quali	^30^

Notes: Upper-MICs, upper-middle-income countries; Lower-MICs, lower-middle-income countries; LICs, low-income countries; –, Not available; Quali, Qualitative research; Quant, Quantitative research.

#### Financing and payment arrangements

Free vaccination through NIPs was provided among 73 countries with data. Co-financing by government and external funding was most common (37 countries): 58.3% (7/12) of LICs, 54.5% (18/33) of lower-MICs, and 30.8% (12/39) of upper-MICs. Government-only financing ranked second, most prevalent among upper-MICs (35.9%). External funding exclusively was third: 24.2% (8/33) of lower-MICs and 25.0% (3/12) of LICs. External sources include Gavi, WHO, UNICEF, UNFPA, PATH, CHAI, and UICC. Two countries employed alternative strategies: Serbia used social health insurance, while Togo combined external funding with social health insurance (Supplemental Figure S6).

### Coordination and partnership mechanisms

Government interdepartmental collaboration, primarily between Ministries of Health and Education (MOH-MOE, 69/84, 82.1%), was universal across all 84 countries. International partnerships, reported in 52 countries (61.9%), varied by income level: GAVI collaborated with 2 upper-MICs, 19 lower-MICs, and 12 LICs. UNICEF and WHO partnerships were more frequent in lower-MICs (11 and 7 countries respectively) and LICs (5 and 3 countries respectively). PAHO collaboration occurred primarily in Latin America. Public-private partnerships were limited to 4 countries (4.8%), and community engagement was documented in 12 countries (14.3%). Multiple collaboration mechanisms commonly coexisted within individual countries (Supplemental Table S5).

### HPV vaccination coverage among adolescents in LMICs

**Fig. 2:**
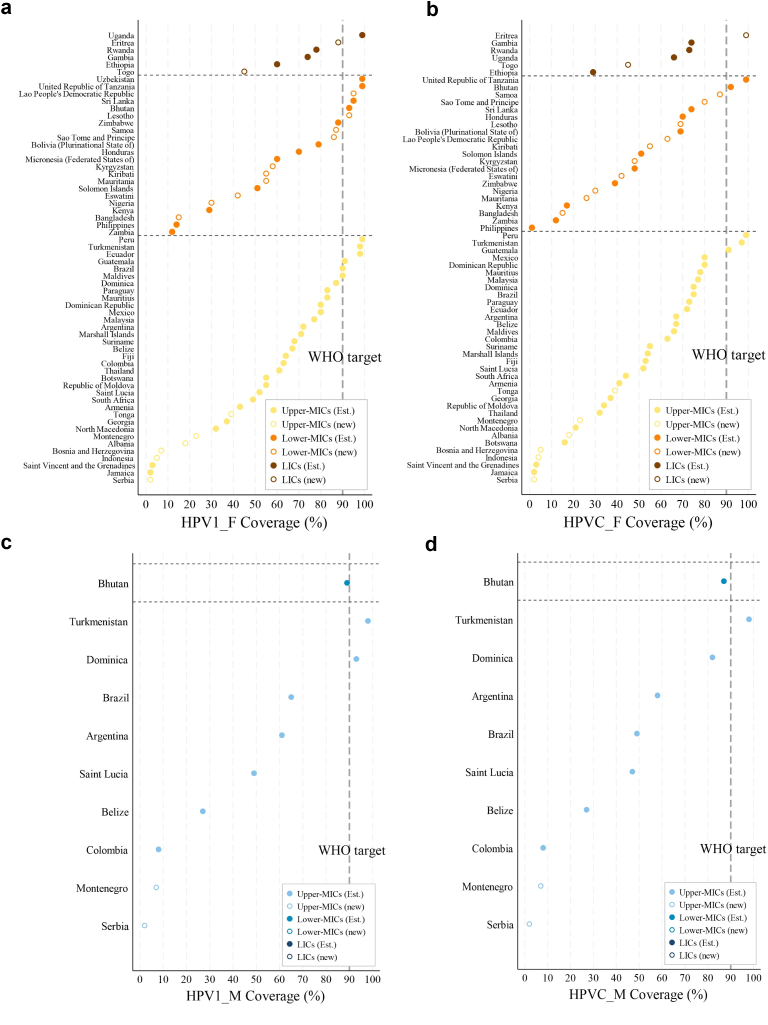
**Scatter plot of HPV vaccination coverage by age 15 in included LMICs**. Notes: Fig. 2a–d show scatter plots of HPV vaccination coverage by age 15 in included low- and middle-income countries (LMICs), corresponding to first dose and last dose among females and males, respectively. Fig. 2a–d presents scatter plots of HPV vaccination coverage by age 15 in included low- and middle-income countries (LMICs), corresponding to 15HPV1_F, 15HPVC_F, 15HPV1_M and 15HPVC_M, respectively. Upper-MICs: upper-middle-income countries; Lower-MICs: lower-middle-income countries; LICs: low-income countries; 15HPV1_F: HPV Vaccination coverage by age 15, first dose, females; 15HPVC_F: HPV Vaccination coverage by age 15, last dose, females; 15HPV1_M: HPV Vaccination coverage by age 15, first dose, males; 15HPVC_M: HPV Vaccination coverage by age 15, last dose, males; Est.: Established countries (≥5 years); new: newly introduced countries (<5 years). HPV vaccination coverage in 84 included countries in 2024 was retrieved from WHO website, 62 countries provided 15HPV1_F data, 61 countries provided 15HPVC_F data, 10 countries provided 15HPV1_M data and 15HPVC_M data.

In 2024, most LMICs remain below the WHO 90% coverage target. Among 62 countries reporting first-dose coverage among females by age 15, only 21.0% (13 countries) achieved the WHO 90% target, while 30.6% (19 countries) remained below 50%. For complete coverage among female by age 15, merely 9.8% (6 countries) reached the 90% target and 44.3% (27 countries) remained under 50%. Male vaccination data were substantially more limited, with only 10 countries reporting (Fig. 2).

LICs generally achieved the highest coverage levels, with several established programs meeting or approaching the WHO 90% target. Lower-MICs fell into a moderate, widely dispersed range, while Upper-MICs exhibited substantial variability, with most clustered at the lower end of the coverage distribution and only a few achieving high uptake.

When distinguishing by program maturity, most LIC programs were established (≥5 years) and concentrated in the high-coverage range. Among lower-MICs, both established and newly introduced programs were spread across the coverage spectrum. Among upper-MICs, newly introduced programs (<5 years) were generally located in the lower-coverage range, while established programs showed wider dispersion, with several established programs remaining below 50%.

Regional disparities were evident (Supplemental Figures S7–S10). Female coverage was highest in Latin America, while Asia and Africa showed more variable performance. Male vaccination data were concentrated in Latin American countries that include boys in their programs.

### Coverage patterns by vaccination policy

To examine how program design influences vaccination performance, we assessed HPV vaccination coverage by key vaccination policy, including delivery platform, dose regimen and rollout mode.

#### Coverage by delivery platform

**Fig. 3:**
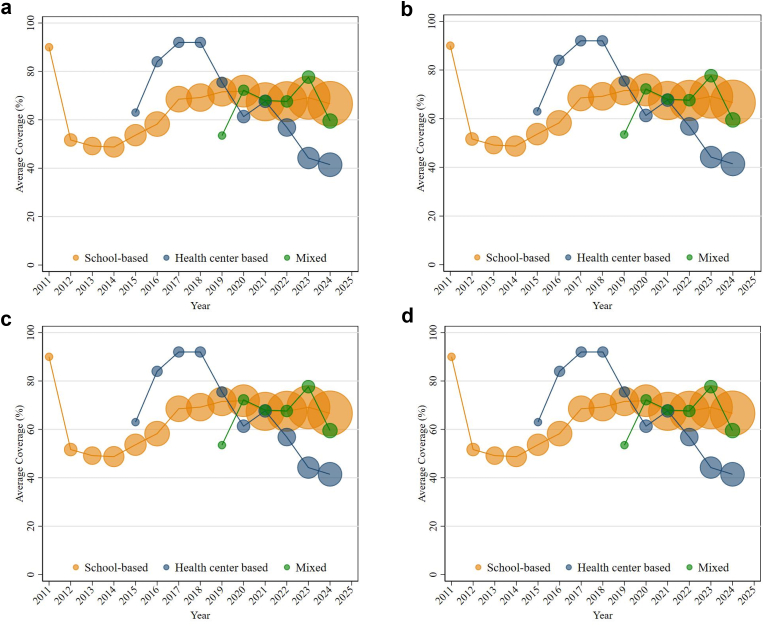
**Trends in HPV vaccination coverage by age 15, grouped by delivery platform (2011–2024)**. Notes: Fig. 3a–d presents trends in HPV vaccination coverage by age 15 across 2011–2024, grouped by delivery platform, corresponding to 15HPV1_F, 15HPVC_F, 15HPV1_M and 15HPVC_M, respectively. Circle size = Number of valid countries (group/year). 15HPV1_F: HPV Vaccination coverage by age 15, first dose, females; 15HPVC_F: HPV Vaccination coverage by age 15, last dose, females; 15HPV1_M: HPV Vaccination coverage by age 15, first dose, males; 15HPVC_M: HPV Vaccination coverage by age 15, last dose, males; HPV vaccination coverage in 84 included countries in 2024 was retrieved from WHO website, 62 countries provided 15HPV1_F data, 61 countries provided 15HPVC_F data, 10 countries provided 15HPV1_M data and 15HPVC_M data.

In countries with school-based programs, we observe more consistent, stabler and higher coverage over time compared to health facility-based approaches. Among females, except for higher coverage in 2011, average first-dose coverage by age 15 in school-based programs remained stable around 60% (range 49%–72%), comparable to mixed approaches but more stable than health facility-based approaches (range 41%–92%). Similar patterns emerged for complete-dose coverage among females by age 15, though with lower absolute values. For males, mixed strategies achieved the highest average first-dose coverage. Between 2018 and 2022, early male HPV vaccination adopters exclusively used health facility-based programs. From 2022 onward, more countries adopted school-based or mixed approaches, with school-based strategies achieving higher coverage than health facility-based strategies after 2023. A similar disparity was observed for complete-dose coverage among males (Fig. 3).

#### Supplementary strategies for reaching underserved adolescents

Supplementary strategies for reaching underserved adolescents. Among 39 countries using school-based programs, 9 (23.1%) documented supplementary strategies for out-of-school girls, most commonly referral to health facilities (7/9), followed by community outreach and multi-component approaches (Supplemental Table S6). Among 15 countries using mixed approaches, 5 (33.3%) reported specific provisions for underserved populations, such as health facilities for remote or vulnerable communities and combined school-health facility-community delivery. Coverage among these countries varied considerably: for school-based programs with supplementary strategies, first-dose coverage ranged from 51% (Solomon Islands) to 95% (Sri Lanka) and complete-dose coverage from 16% (Botswana) to 80% (Mexico); among mixed-strategy countries, Eritrea achieved high coverage (99%) while Bangladesh reported substantially lower rates (15%).

#### Coverage by dose regimen

First-dose coverage was broadly comparable across regimens. Among females, the three-dose schedule (introduced in 2011) sustained stable first-does coverage of 63%–79% during 2013–2023; as programs evolved, most countries transitioned from three-dose to two-dose and then to single-dose schedules. At present, only a small number of countries retain the three-dose schedule with generally low coverage. Two-dose adoption expanded from 2014, with coverage stabilizing at 66%–73% from 2017 onwards. Single-dose schedules emerged in 2020 and were increasingly adopted after 2022, reaching 61% in 2024, similar to the 66% under two-dose schedules. Among males, no country adopted a three-dose schedule given the later launch of male vaccination; two-dose schedules rose alongside the growing number of implementing countries from 2018 and the first-dose coverage stabilized around 60%, whereas single-dose schedules, adopted since 2023, showed lower coverage (44%–55%) (Supplemental Figure S11).

#### Coverage by rollout mode

Full-swing rollout strategies yielded more stable coverage trajectories than stepwise approaches, though both have declined recently. Among females, average first-dose coverage under full-swing strategies remained stable around 60%, showing greater stability than stepwise strategies. Average complete-dose coverage among females followed a comparable pattern with lower absolute values. Among males, stepwise strategies maintained relatively stable average first-dose coverage around 65%, while full-swing strategies showed slightly lower and declining coverage. Similar disparities existed for average complete-dose coverage among males (Supplemental Figure S12).

### Evidence on HPV vaccination implementation in LMICs

The available literature showed that HPV vaccination implementation in LMICs had achieved meaningful progress, supported by governmental leadership, intersectoral collaboration, and targeted capacity building. However, implementation remained challenged by system and resource constraints, socio cultural and knowledge barriers, vaccine hesitancy, and persistent equity gaps, highlighting uneven progress across countries and income groups.

### Achievements

Studies from 15 countries (6 upper-MICs,^16^, ^17^, ^18^, ^19^, ^20^, ^21^ 7 lower-MICs,^22^, ^23^, ^24^, ^25^, ^26^, ^27^, ^28^ and 2 LICs^29^^,^^30^) provided implementation experiences covering four key aspects: policy and institutional design (governance, leadership, policy innovation); service delivery and collaboration (delivery models, partnership mechanisms); communication strategies (information dissemination, publicity channels); and capacity building (personnel training, electronic systems, technology transfer, financing mechanisms) (Table 2).

Upper-MICs leveraged strong governmental leadership and policy innovation supported by robust technological infrastructure: Argentina^16^ coordinated through its National Department of Health with provincial adaptation (reaching 72% first-dose coverage among females in 2024), Brazil^17^ established an electronic registry and technology-transfer mechanisms (90%), the Dominican Republic^18^ paired an affordability policy with multi-channel publicity (80%). Lower-MICs prioritized multisectoral, particularly health-education, collaboration (Zimbabwe^28^ and Tanzania,^27^ with 88% and 99% coverage, respectively), complemented by digital monitoring systems (Bhutan,^22^ 93%). LICs focused on strategic planning with strong political commitment and external partnerships (Rwanda,^29^ 78%) and on culturally adapted community engagement to reach vulnerable populations, exemplified by Mali's^30^ community health-worker sessions and storytelling-cloth approach. Across income levels, these higher-coverage programs were generally built on a stable school-based or mixed platform reinforced by intersectoral collaboration, affordability measures, and robust monitoring or registry systems (Table 2 and Fig. 2).

### Barriers

Studies from 17 countries (3 upper-MICs,^21^^,^^31^^,^^32^ and 11 lower-MICs,^22^^,^^25^^,^^26^^,^^28^^,^^33^^,^^34^ and 3 LICs^33^) reported implementation barriers categorized into four types: system barriers (policy implementation and institutional management challenges), sociocultural barriers (societal attitudes, cultural beliefs, media influence), knowledge barriers (awareness and information access limitations), and resource barriers (human, material, and financial constraints). Upper-MICs predominantly encountered sociocultural and knowledge barriers, including vaccine safety concerns, fertility-related fears, and social media misinformation. Lower-MICs and LICs faced more fundamental resource constraints, including supply chain vulnerabilities, high costs, transportation barriers, and inadequate infrastructure (Supplemental Table S7).

### Vaccination hesitancy

Studies from 7 countries^18^^,^^25^^,^^34^, ^35^, ^36^, ^37^, ^38^ have reported detailed hesitancy reasons. Among the reported causes, safety concerns, lack of knowledge, and socio-cultural factors are the most common. Religious beliefs and sexual behavior concerns emerged as prominent hesitancy drivers in lower-MICs, while upper-MICs more frequently reported accessibility and cost-related barriers alongside safety concerns. No LICs reported hesitancy data (Supplemental Table S8).

### Equity

Limited evidence from 2 countries^32^^,^^39^ revealed equity challenges including educational disparities (in-school versus out-of-school girls), geographic inequities (urban-rural gaps and marginalized area prioritization), and vulnerabilities among specific populations such as ethnic minorities and HIV-positive individuals (Supplemental Table S9).

## Discussion

This scoping review provides the first comprehensive analysis of adolescent HPV vaccination strategies and implementation across 84 LMICs that have incorporated HPV vaccines into their NIPs. Our findings reveal policy heterogeneity and diverse implementation experiences that offer critical insights for optimizing adolescent HPV vaccination programs in resource-constrained settings.

### HPV vaccination policy landscape across LMICs

Regarding HPV introduction, upper-MICs led adoption with Latin America as the regional pioneer, while Asia and Africa lagged behind. Policy contents demonstrated both convergence and divergence across income levels. Convergence was observed in target age (9–14 years as the primary group), delivery platform (school-based approach dominant at 66.7% in both upper-MICs and lower-MICs), and free vaccination provision. A clear trend toward dosing simplification emerged, with nearly half of LMICs now adopting single-dose schedule. Key divergences persisted in gender eligibility, with the vaccination of boys largely confined to upper-MICs (51.3%), and in vaccine type, nonavalent vaccines exclusively adopted by upper-MICs, bivalent use concentrated in lower-income settings. The correlation between vaccine valency and gender eligibility is likely reflects the differential cost-effectiveness implications, as bivalent vaccines offer limited benefit rationale for male vaccination, while higher-valency products provide broader protection against HPV-related disease in both sexes.^40^ In financing mechanisms, LICs relying heavily on external support. Cooperation mechanisms universally featured government interdepartmental collaboration (MOH-MOE), while international partnerships and community engagement varied by income level. Despite these policy advances, coverage remained suboptimal-only 9.8% of countries achieved the WHO 90% complete vaccination target in female. Notably, LICs generally achieved the highest levels, likely reflecting strong Gavi support and concentrated programmatic investment,^41^ while upper-MICs exhibited wide variability, with several established programs (≥5 years) remaining below 50%, indicating that program duration and income level alone do not guarantee high coverage. Coverage analysis revealed that school-based vaccination programs maintained more stable performance over time, and simplified dosing schedules does not compromise a program's ability to reach adolescents with an initial vaccination.

### Implementation experiences and barriers

Evidence from 15 countries identified four key success factors: robust policy and institutional design, effective service delivery and collaboration, tailored communication strategies, and sustained capacity building. Conversely, 17 countries reported implementation barriers varying by income level, upper-MICs predominantly faced social-cultural and knowledge barriers, while lower-MICs and LICs encountered more fundamental resource constraints. Vaccine hesitancy, documented in 7 countries, was primarily driven by safety concerns, knowledge deficits, and socio-cultural factors. Equity challenges, though limited to 2 countries, revealed disparities across educational status, geographic location, and vulnerable populations.

In contrast to high-income countries such as Australia^42^ and Scotland,^43^ where school-based HPV vaccination achieved >80% and >90% coverage respectively through well-established health systems and comprehensive financing, LMICs face fundamentally different contexts characterized by resource constraints and structural barriers.^5^^,^^44^ These disparities necessitate context-specific strategies.

### Core elements of successful strategies

#### School-based vaccination program and mixed model adaptability

School-based programs have been widely adopted in LMICs (66.7% of upper-MICs, 66.7% of lower-MICs, 41.7% of LICs). Coverage trend analysis shows average female first-dose coverage using school-based programs remained stable around 60%, comparable to mixed approaches but more stable than health facility-based programs. For males, school-based programs achieved higher coverage than health facility-based strategies since 2023. School-based program success stems from several advantages: centralized access to target populations leveraging high enrollment rates for economies of scale^44^; peer support in school environments alleviating vaccination anxiety^45^; and elimination of additional household expenditure and time burdens, improving acceptance.^46^

However, this stable average coverage may mask the systematic exclusion of out-of-school adolescents.^47^ Only 23.1% of school-based programs documented supplementary strategies for unschooled girls, predominantly referral to health facilities. The wide coverage variation among these countries suggests that having a supplementary strategy is insufficient. Sri Lanka (95%) and Botswana (55%), both using health facility supplementation, illustrate that identical approaches can yield vastly different equity outcomes depending on health system capacity. This concern is particularly acute in low-income countries where out-of-school girls are most concentrated.^48^ Mixed delivery models offer a more structurally inclusive framework: Eritrea's combined approach with provisions for remote villages achieved 99% last-dose coverage, while Mozambique and Mali demonstrated the potential of multi-component models integrating mobile outreach, community health workers, and culturally adapted communication to reach marginalized groups.^30^ For LMICs, policy focus should extend beyond selecting a primary delivery platform to explicitly designing supplementary strategies with attention to implementation quality.

#### Simplified dosing schedules

The evolution toward simplified dosing schedules reflects an evidence-based policy optimization process. Our findings documented a clear temporal progression from universal three-dose schedules (2008–2013) to predominant two-dose adoption (2014–2017), culminating in widespread single-dose implementation beginning since 2023. Currently, nearly half of all included LMICs have adopted single-dose schedules.

Our data show that in 2024, mean first-dose coverage among female populations was 61% under single-dose programs and 66% under two-dose programs, with no substantial difference between them. This suggests that reducing the number of required doses does not compromise a program's ability to reach adolescents with an initial vaccination. Meanwhile, multi-dose programs consistently experience inter-dose attrition, resulting in full-course coverage rates lower than first-dose coverage. Single-dose schedules also reduce costs associated with subsequent doses, ease cold-chain requirements, and simplify logistics, thereby lowering overall implementation burden.^49^ The WHO's 2022 single-dose recommendation, based on emerging evidence of sustained immunogenicity and effectiveness,^50^ offering LMICs a potentially more feasible pathway to overcoming implementation barriers. Although an additional dose is sometimes regarded as a further contact point that could re-engage adolescents who missed earlier vaccination, the comparable first-dose coverage across regimens and the persistent inter-dose attrition under multi-dose schedules indicate that this benefit does not materialize at the population level. Where coverage remains low or inequitably distributed, the priority should be strengthening service delivery systems and optimizing catch-up strategies (e.g., periodic outreach rounds, hybrid delivery models) rather than retaining additional doses.

In summary, adopting single-dose schedules represents a meaningful programmatic simplification for LMICs that may help resource-constrained countries expand effective immunization coverage more efficiently.

#### Additional success factors in high-performing countries

High-performing countries also demonstrated the following four critical implementation elements: robust policy and institutional design, effective service delivery and collaboration mechanisms, comprehensive communication strategies, and sustained capacity building efforts.

#### Policy and institutional design

Successful countries strengthened governance and leadership structures and promoted policy innovation. Argentina and South Africa emphasized strong national-level leadership and coordination,^16^^,^^17^ Rwanda relied on detailed planning and firm political commitment,^29^ while Serbia^20^ and the Dominican Republic^18^ addressed challenges through localized innovations such as pharmacy-based health education and affordability policies. By adapting to local resources, health system characteristics, and sociocultural contexts, these designs addressed leadership coordination bottlenecks and established sustainable support systems for long-term coverage improvement.

#### Service delivery and collaboration

Multi-sectoral cooperation mechanisms were key to success. Most countries emphasize health-education sector collaboration, facilitated successful school-based programs. International partnerships, especially Gavi support, played a critical role in advancing program implementation in Lower-MICs and LICs.^41^

#### Communication strategies

Communication strategies improving vaccine acceptance.^51^ Cameroon^24^ and Tanzania^27^ have adopted mass media and social media approaches, while Mali has utilized culturally adapted storytelling methods^30^ These contextually tailored communication practices facilitate the better local rollout of HPV vaccines.

#### Capacity building

Successful countries invested in personnel training, electronic systems, and financing guarantees. Cambodia^23^ and Kenya^25^ have conducted training for teachers and healthcare workers, Bhutan^22^ has developed digital monitoring and reporting systems and Brazil has established electronic registration systems and technology transfer mechanisms.^17^ These measures have strongly supported the implementation of policies.

### Implementation challenges

Despite progress, adolescent HPV vaccination in LMICs faces multifaceted challenges. Resource barriers represent the most fundamental constraint, including high vaccine costs, supply chain vulnerabilities, transportation challenges, and inadequate human resources. Countries such as Uzbekistan,^34^ Kenya,^25^ and Nigeria^26^ identified cost and accessibility as primary impediments, creating substantial programmatic vulnerabilities particularly in low-income settings with competing health priorities and limited fiscal space. Sociocultural barriers in upper-MICs, included vaccine hesitancy related to safety concerns, fertility anxieties, and religious objections, requiring adapted communication strategies that address cultural sensitivities while maintaining scientific accuracy.^52^ Knowledge barriers, manifesting as limited public awareness and healthcare worker knowledge gaps, necessitate comprehensive educational interventions targeting multiple stakeholder groups.

Vaccine hesitancy patterns revealed consistent themes, with safety concerns, knowledge deficits, and sociocultural factors as primary drivers. The prominence of misinformation, particularly through social media, highlights the need for proactive communication strategies that counter false narratives while building public confidence.^53^ Equity issues revealed multidimensional disparities including educational inequities (in-school versus out-of-school girls), geographic imbalances,^54^ and vulnerabilities among ethnic minorities and HIV-positive individuals.^39^ These findings underscore the need for inclusive vaccination strategies extending beyond school-based platforms to reach marginalized populations.

This divergence likely reflects both genuine contextual differences and the limits of the available evidence. Upper-MICs generally have stronger health systems, financing, and infrastructure, so foundational supply-side barriers are largely resolved and the binding constraints shift toward demand-side factors such as hesitancy^52^ and misinformation, the latter amplified by higher social-media penetration.^53^ In LICs and many lower-MICs, by contrast, weaker infrastructure and reliance on external funding keep supply-chain, logistical, and human-resource constraints predominant.^46^ These patterns warrant caution, however, as barrier evidence came from few, unevenly distributed countries; the barriers reported partly mirror the research conducted, with demand-focused studies more common in better-resourced settings; and sparse LIC evidence may understate resource challenges where they are most acute.

### Implications for policy and practice

The evidence synthesized in this review provides clear direction for optimizing adolescent HPV vaccination strategies in LMICs.

Countries may consider prioritizing WHO-recommended single-dose schedules as a logistically feasible option for broadening coverage, while establishing robust monitoring systems to evaluate long-term protection.

In many contexts, school-based approaches represent a cost-effective option and can serve as a preferred delivery model, supplemented by mixed strategies to reach out-of-school adolescents, remote populations, ethnic minorities, and HIV-positive youth^46^; however, the optimal delivery design is context-specific and countries need to define tailored evaluation criteria and routinely analyze relevant indicators to refine delivery.

LICs may generally prioritize improving girls' vaccination coverage; MICs could consider extending vaccination to boys after achieving high female coverage (e.g.,≥80%), while such expansion requires careful assessment of local epidemiological needs, available funding, and cost-effectiveness.^55^ Countries with strong local justification and sustainable resources may reasonably extend routine vaccination to both boys and girls regardless of income level.

Given the near-absence of ICP-specific vaccination policies in lower-MICs and LICs, clearer international guidance is needed to support these countries in developing evidence-based strategies for immunocompromised populations.

Communication strategies must be adolescent-friendly, incorporating age-appropriate and culturally adapted materials,^51^^,^^56^ leveraging social media while countering misinformation, and implementing service standards that respect adolescent autonomy while ensuring parental involvement.

### Strengths and limitations

This scoping review possesses several notable strengths. First, focusing on adolescents aged 9–14 years provides targeted insights for the most cost-effective cervical cancer prevention approach. Second, this represents the first systematic documentation of adolescent HPV vaccination strategies across all 84 LMICs, utilizing multiple sources for comprehensive policy capture. Third, systematically tracking temporal evolution enables identification of policy transition patterns, providing policymakers crucial context for understanding rationale and feasibility. Fourth, stratifying coverage data by implementation strategy across multiple years provides critical evidence for evaluating sustained performance, which is particularly valuable for LMICs designing or modifying programs.

Several limitations warrant acknowledgment. First, incomplete reporting of coverage data from LMICs to WHO (Unreported rate of 26%–27% for females and 58% for males) may introduces selection bias. Countries with weaker health systems and lower coverage are likely underrepresented among reporting countries, suggesting that observed coverage levels may overestimate true population-level performance. Second, implementation experience and barrier documentation were particularly sparse for LICs, limiting the generalizability of findings to settings where implementation challenges are likely most severe. Third, heterogeneity in reporting standards precluded quantitative synthesis, making conclusions tentative and hypothesis-generating rather than definitive. Fourth, national-level focus may overlook important local variations contributing to implementation success. Fifth, although a predefined review protocol guided the study process, the absence of protocol registration may have reduced the transparency and reproducibility of the review process. Sixth, cross-strategy coverage comparisons were unadjusted for potential confounders (e.g., GDP, health system capacity, programme duration, Gavi eligibility), as this scoping review was not designed as a controlled analysis; observed differences in coverage stability therefore reflect associations rather than attributable effects of delivery strategy. Seventh, the implementation literature may be subject to publication bias, as countries with successful programmes are more likely to publish their experiences, potentially overrepresenting enabling factors relative to structural barriers. Additionally, selecting a single primary reference per country may additionally result in some loss of longitudinal implementation information.

### Conclusions

Adolescent HPV vaccination in LMICs presents both significant challenges and substantial opportunities for cervical cancer prevention. While coverage remains suboptimal across most countries, successful implementations demonstrate that resource constraints need not be insurmountable barriers. School-based programs paired with complementary mixed approaches constitute a promising option to advance equitable population-level protection, though the most suitable delivery model varies by local conditions after systematic programme evaluation. The WHO's single-dose recommendation offers LMICs a practical opportunity to simplify implementation and reduce costs. Success also requires robust policy frameworks, effective service delivery and collaboration, innovative communication strategies, and sustained capacity building. This review provides a foundation for evidence-informed policy development in LMICs. With strategic focus and sustained effort, LMICs can substantially improve adolescent HPV vaccination coverage and contribute meaningfully to the WHO's 2030 cervical cancer elimination goals.

Future research should prioritize implementation science studies comparing school-based versus mixed-method delivery strategies in LMIC contexts. Long-term effectiveness studies of single-dose vaccination remain essential for confirming public health.^57^ Research on adolescent-specific factors affecting vaccine acceptance, including peer influences and culturally appropriate communication strategies, would provide valuable guidance. Studies investigating approaches for reaching out-of-school adolescents and marginalized populations could address equity gaps.

## Contributors

Shudan Liu: Conceptualization, Information search, Screening, Data extraction, Data synthesis, Writing-Original Draft, Writing-Review & Editing; Di Wu: Conceptualization, Information search, Screening, Data extraction, Writing-Original Draft; Siyan Ye: Information search, Screening, Data extraction; Yuqing Xiao: Screening, Data extraction; Meng Wu: Screening, Data extraction; Shu Chen: Conceptualization, Writing-Review & Editing; Xinyu Zhang: Conceptualization, Writing-Review & Editing; Lei Guo: Writing-Review & Editing; Qin Zhang: Information search, Screening, Data extraction; Yang Zhou: Screening, Data extraction; Xiangju Wu: Screening, Data extraction; Qian Yang: Screening, Data extraction; Rongchang Xiao: Screening, Data extraction; Jie Zhang: Information search; Xinyi Feng: Information search; Jinghan Wang: Information search, Screening, Data extraction; Xianwen Shi: Information search, Screening, Data extraction; Xiaoyuan Zhang: Screening, Data extraction; Jiawei Xu: Writing-Review & Editing; Jianchao Shao: Information search, Data extraction; Shenglan Tang: Conceptualization, Writing-Review & Editing, Supervision; Qin Liu: Conceptualization, Data synthesis, Writing-Review & Editing, Supervision. All authors read and approve the final version of the manuscript. Qin Liu had final responsibility for the decision to submit for publication.

## Data sharing statement

The data used in this study are extracted from published articles, official reports and official websites as indicated in the main text and references.

## Editor note

The Lancet Group takes a neutral position with respect to territorial claims in published maps and institutional affiliations.

## Declaration of interests

We declare no competing interests.
