# Corrigendum to “HPV vaccination policies and implementation for adolescents in low- and middle-income countries: a scoping review” [The Lancet Regional Health-Western Pacific Volume 72, July (2026), 101919]

**DOI:** 10.1016/j.lanwpc.2026.101967

**Published:** 2026-09-16

**Authors:** Shudan Liu, Di Wu, Siyan Ye, Yuqing Xiao, Meng Wu, Shu Chen, Xinyu Zhang, Lei Guo, Qin Zhang, Yang Zhou, Xiangju Wu, Qian Yang, Rongchang Xiao, Jie Zhang, Xinyi Feng, Jinghan Wang, Xianwen Shi, Xiaoyuan Zhang, Jiawei Xu, Jianchao Shao, Shenglan Tang, Qin Liu

**Affiliations:** a Research Center for Environment and Human Health, Research Center for Medicine and Social Development, School of Public Health, Chongqing Medical University, Chongqing, China; b School of Nursing, Chongqing Medical University, Chongqing, China; c National Centre for Immunisation Research and Surveillance, New South Wales, Australia; d Duke Global Health Institute, Duke University, NC, USA; e Global Health Research Center, Duke Kunshan University, Kunshan, Jiangsu, China; f Chongqing Center for Disease Control and Prevention (Chongqing Academy of Preventive Medicine), Chongqing, China; g Immunization Program Management Department, Chongqing Shapingba District Center for Disease Control and Prevention, Chongqing, China

The authors regret that the published version of “Fig. 3. Trends in HPV vaccination coverage by age 15, grouped by delivery platform (2011–2024)” contained an error. Fig. 3 combines coverage trend panels for four indicators-15HPVC_F, 15HPV1_M, 15HPVC_M, and 15HPV1_F. Due to an error during figure preparation, the panels intended to show 15HPVC_F, 15HPV1_M, and 15HPVC_M were all mistakenly replaced with the panel for 15HPV1_F. The corrected Fig. 3 is provided with this corrigendum. All other results, tables, and text in the article remain unchanged.

The authors would like to apologise for any inconvenience caused.

**Fig. 3:**
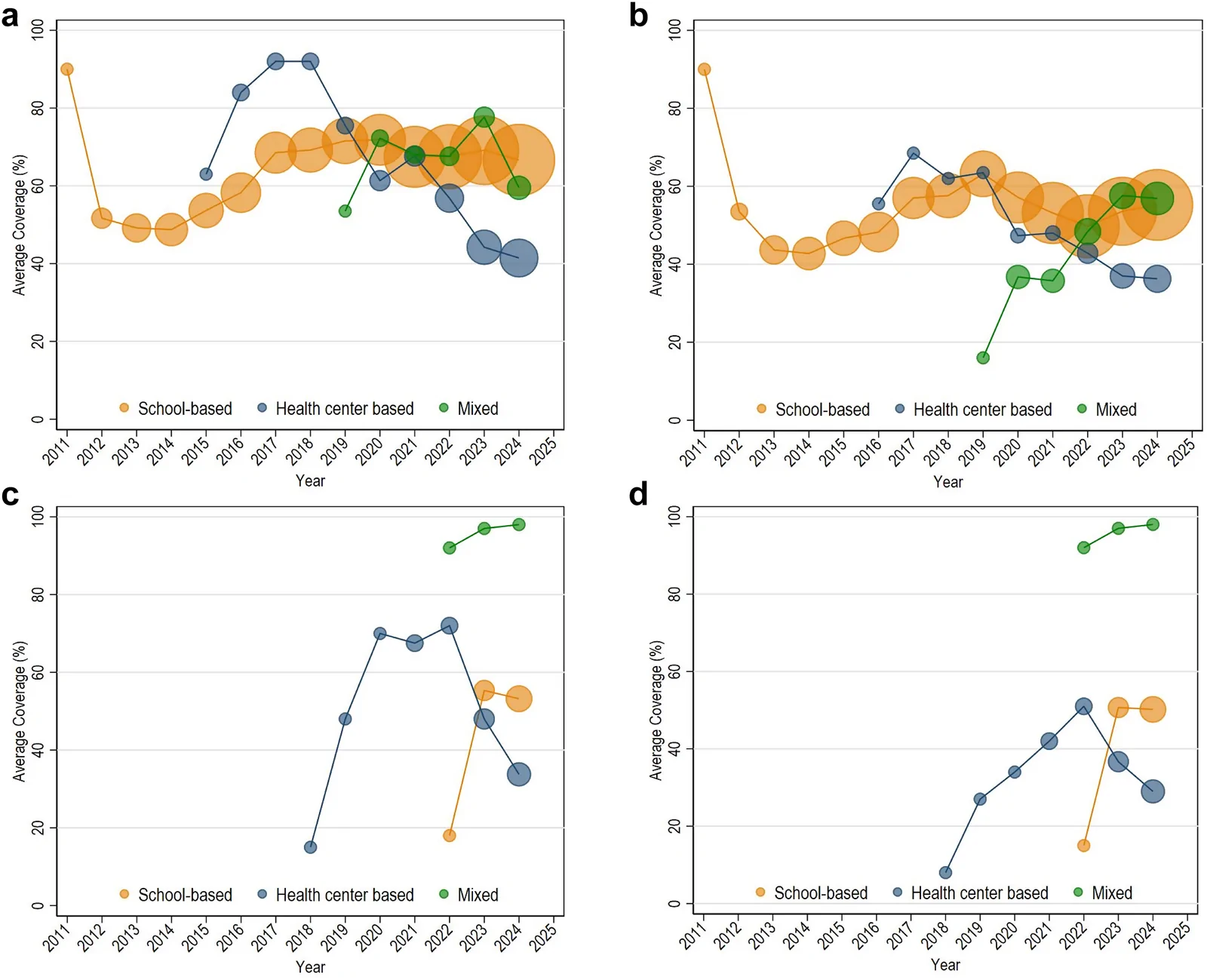
**Trends in HPV Vaccination Coverage by Age 15, Grouped by Delivery platform (2011–2024).** Notes: Figure 3a–d presents trends in HPV vaccination coverage by age 15 across 2011–2024, grouped by delivery platform, corresponding to 15HPV1_F, 15HPVC_F, 15HPV1_M and 15HPVC_M, respectively. Circle size = Number of valid countries (group/year). 15HPV1_F: HPV Vaccination coverage by age 15, first dose, females; 15HPVC_F: HPV Vaccination coverage by age 15, last dose, females; 15HPV1_M: HPV Vaccination coverage by age 15, first dose, males; 15HPVC_M: HPV Vaccination coverage by age 15, last dose, males; HPV vaccination coverage in 84 included countries in 2024 was retrieved from WHO website, 62 countries provided 15HPV1_F data, 61 countries provided 15HPVC_F data, 10 countries provided 15HPV1_M data and 15HPVC_M data.

