# Supplementary material for: HPV vaccination policies and implementation for adolescents in low- and middle-income countries: a scoping review
Source: Lancet Reg Health West Pac. 2026 Jul 8;72:101919. doi: 10.1016/j.lanwpc.2026.101919 (PMC13356746; doi:10.1016/j.lanwpc.2026.101919)
Supplement: Supporting Information [file mmc1.pdf]

S1 File. Supporting Information

Content

Supplementary Figure 1. Literature Screening Process 2
Supplementary Figure 2. HPV Vaccination Gender Coverage by Income Group 3
Supplementary Figure 3. HPV Vaccination Dose Regimen by Income Group 4
Supplementary Figure 4. HPV Vaccine Type Adopted by Income Group 5
Supplementary Figure 5. HPV Vaccination Delivery Platform by Income Group 6
Supplementary Figure 6. HPV Vaccination Funding sources by Income Group 7
Supplementary Figure 7. Map of HPV vaccination coverage by age 15, first dose, females in 84 LMICs 8
Supplementary Figure 8. Map of HPV vaccination coverage by age 15, last dose, females in 84 LMICs 9
Supplementary Figure 9. Map of HPV vaccination coverage by age 15, first dose, males in 84 LMICs 10
Supplementary Figure 10. Map of HPV vaccination coverage by age 15, last dose, males in 84 LMICs 11
Supplementary Figure 11. Trends in HPV Vaccination Coverage by Age 15, Grouped by Dose Regimen (2011-2024) 12
Supplementary Figure 12. Trends in HPV Vaccination Coverage by Age 15, Grouped by Rollout Mode (2011-2024) 13
Supplementary Table 1. Preferred Reporting Items for Systematic reviews and Meta-Analyses extension for Scoping Reviews (PRISMA-ScR) Checklist 14
Supplementary Table 2. Search Strategy Across Different Databases 17
Supplementary Table 3. Evolution process of adolescent HPV vaccination strategies in 84 LMICs18
Supplementary Table 4. Timeline of HPV Vaccine Strategy Adoption into National Immunization Programs in LMICs 45
Supplementary Table 5. Summary characteristics of cooperation mechanism 46
Supplementary Table 6. Supplementary strategies for reaching underserved adolescents and corresponding 2024 HPV vaccination coverage 49
Supplementary Table 7. Summary characteristics of implementation barriers 50
Supplementary Table 8. Summary characteristics of vaccine hesitancy 52
Supplementary Table 9. Summary characteristics of equity 53

Supplementary Figure 1. Literature Screening Process

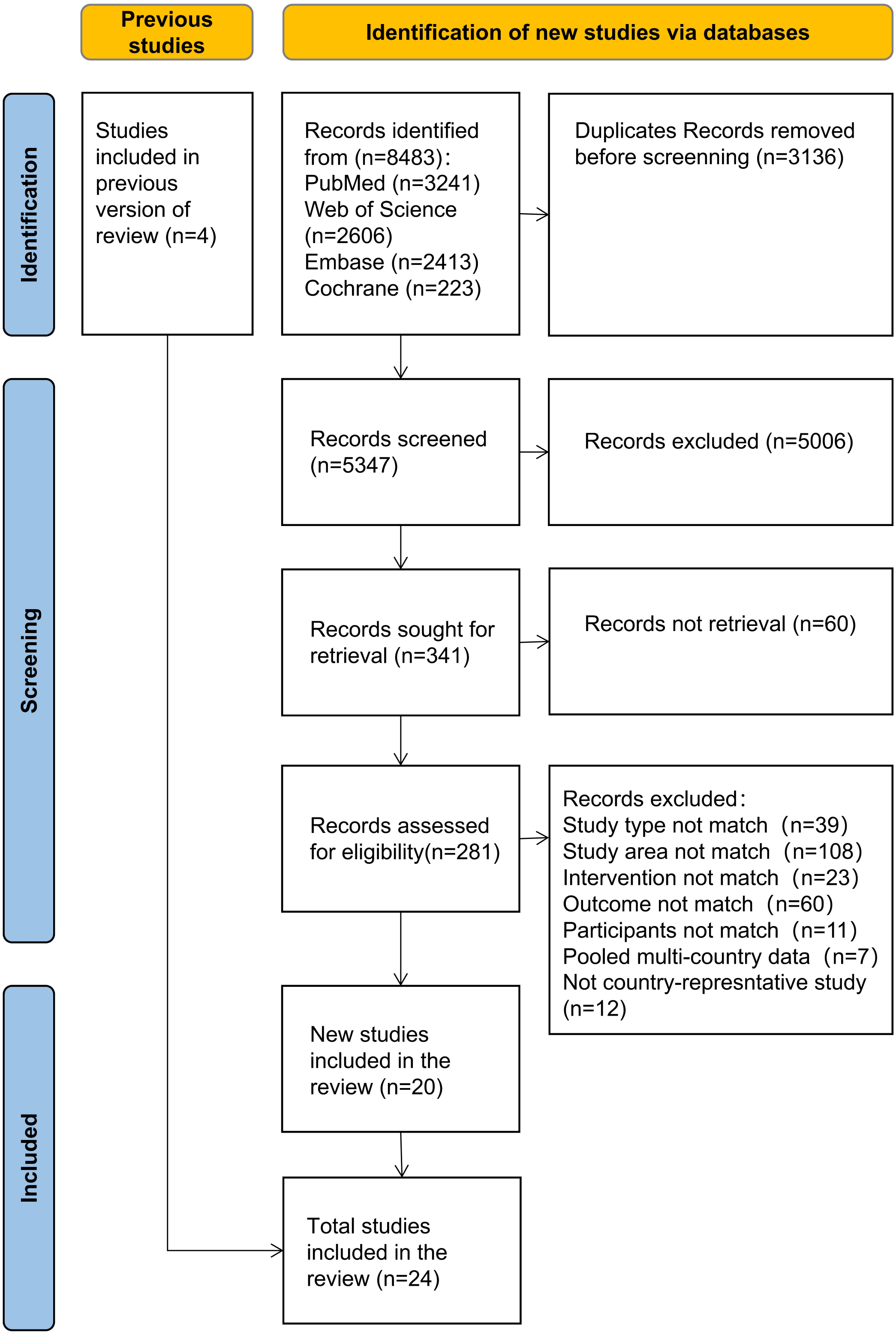

Supplementary Figure 2. HPV Vaccination Gender Coverage by Income Group

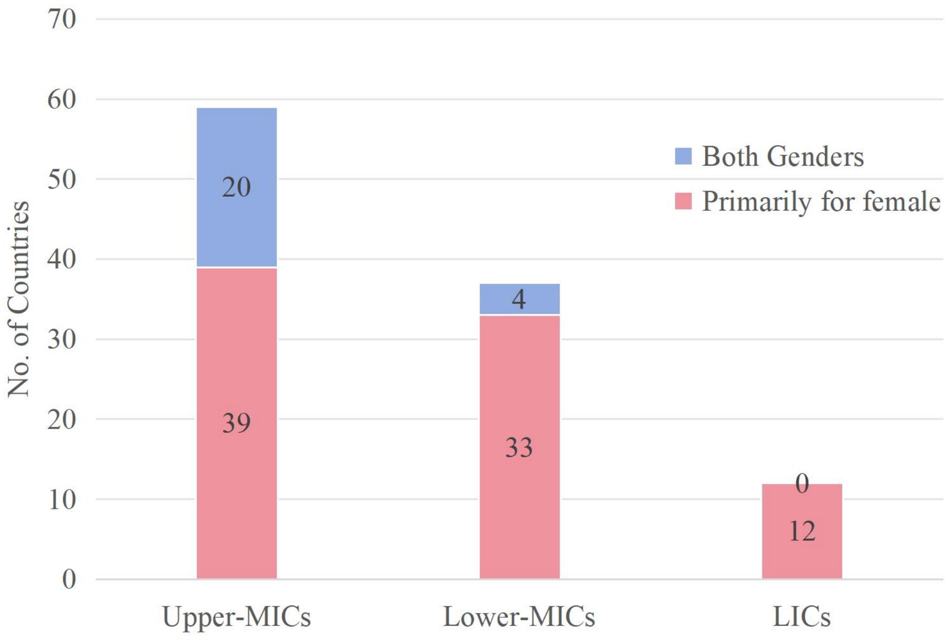

Notes:Upper-MICs:upper-middle-income countries; Lower-MICs:lower-middle-income countries;LICs:low-income countries.

Supplementary Figure 3. HPV Vaccination Dose Regimen by Income Group

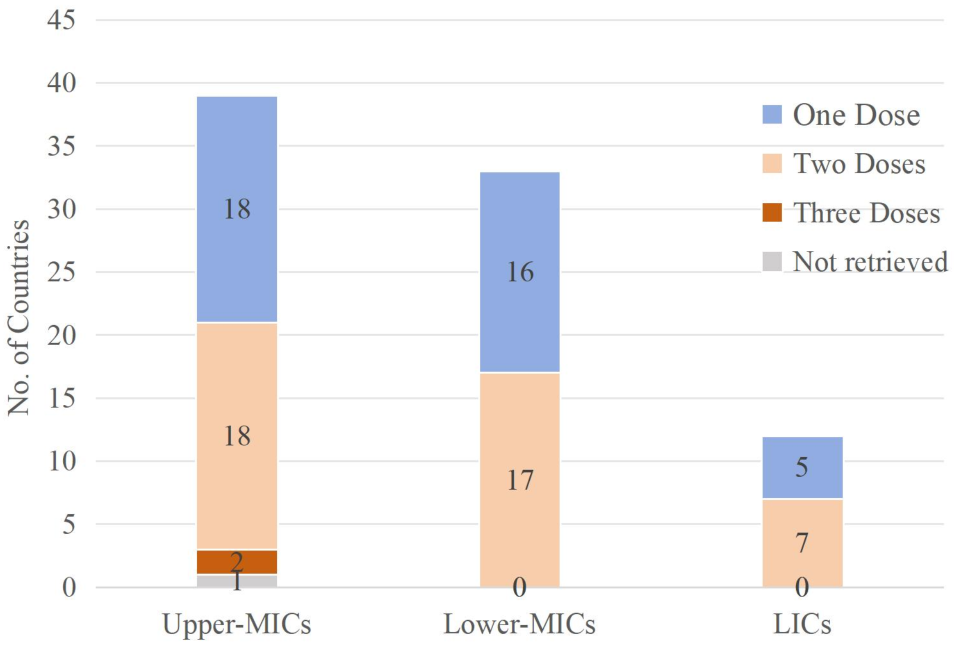

Notes:Upper-MICs:upper-middle-income countries; Lower-MICs:lower-middle-income countries;LICs:low-income countries.

Supplementary Figure 4. HPV Vaccine Type Adopted by Income Group

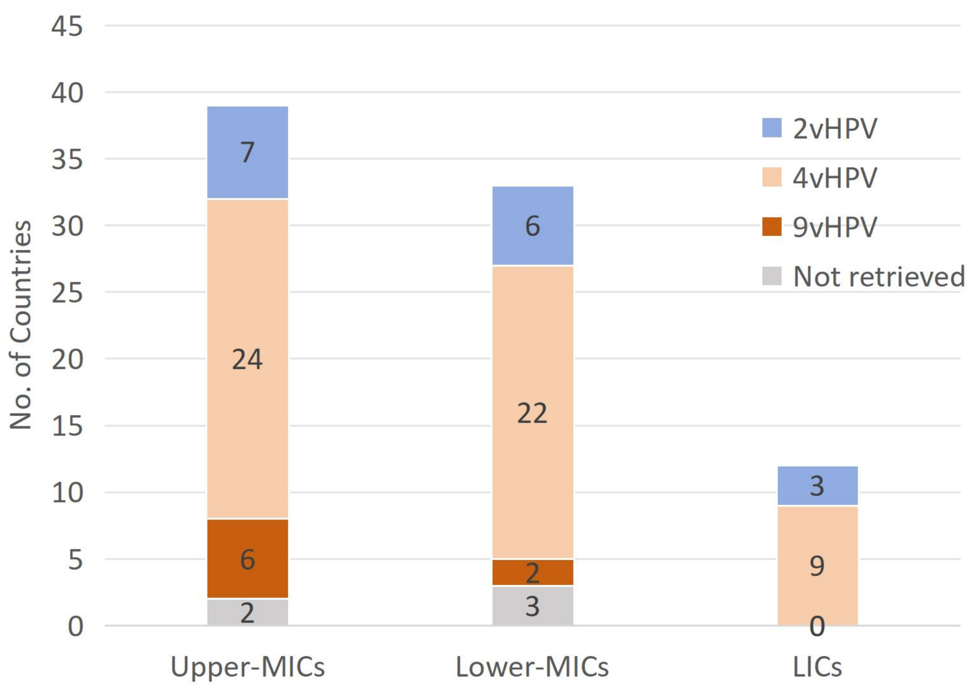

Notes:Upper-MICs:upper-middle-income countries; Lower-MICs:lower-middle-income countries;LICs:low-income countries. 2vHPV:Bivalent Human Papillomavirus Vaccine; 4vHPV:Quadrivalent Human Papillomavirus Vaccine; 9vHPV:Nonavalent Human Papillomavirus Vaccine.

Supplementary Figure 5. HPV Vaccination Delivery Platform by Income Group

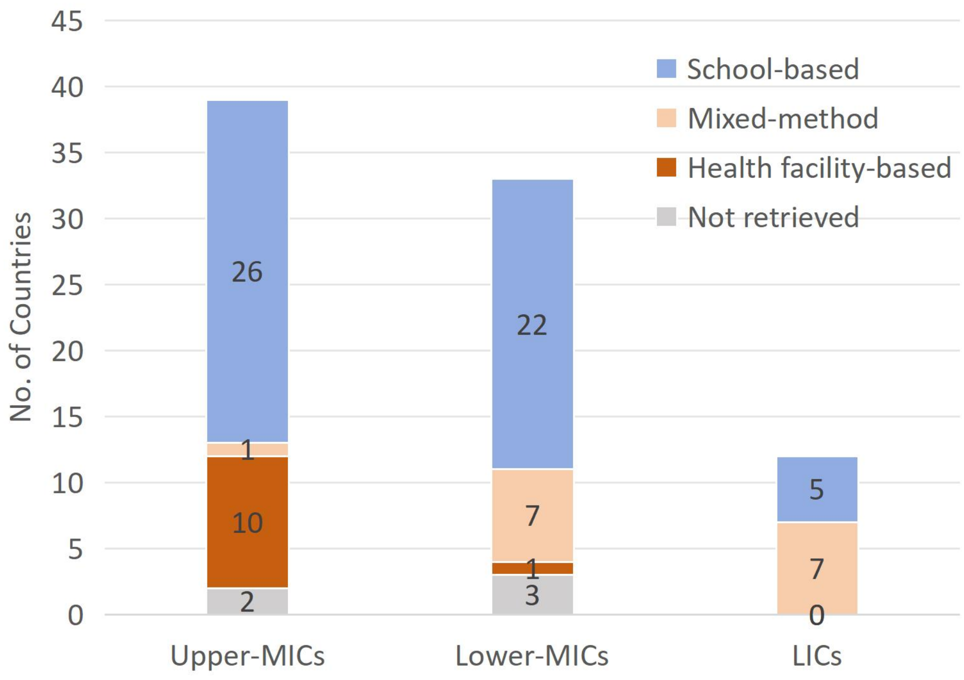

Notes:Upper-MICs:upper-middle-income countries; Lower-MICs:lower-middle-income countries;LICs:low-income countries.

Supplementary Figure 6. HPV Vaccination Funding sources by Income Group

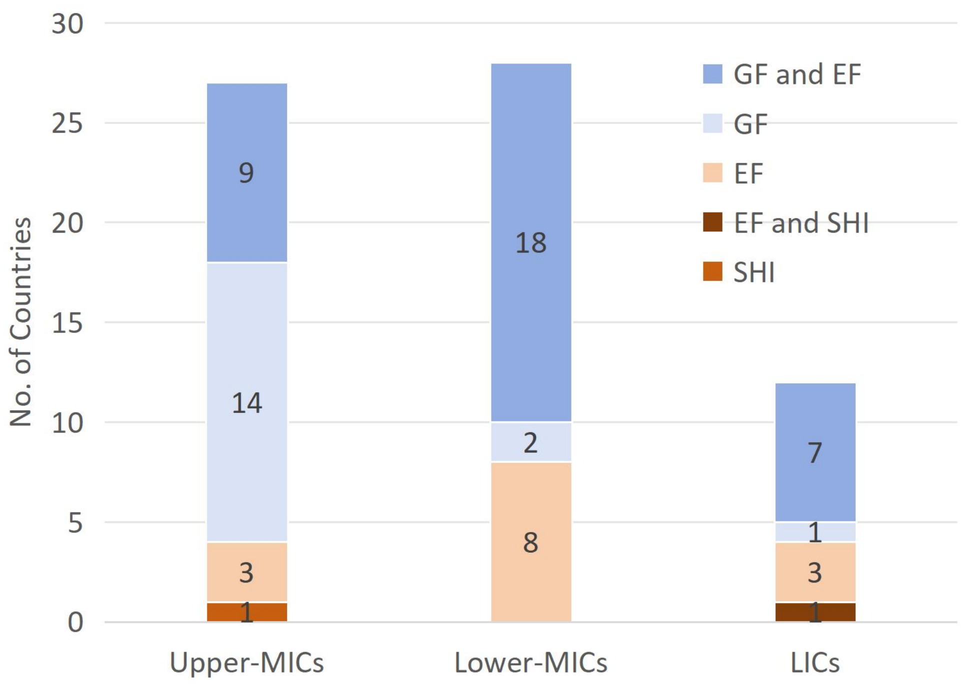

Notes:Upper-MICs:upper-middle-income countries; Lower-MICs:lower-middle-income countries;LICs:low-income countries.GF:Goverment fund; EF:External fund;SHI:Social health insurance.

Supplementary Figure 7. Map of HPV vaccination coverage by age 15, first dose, females in 84 LMICs

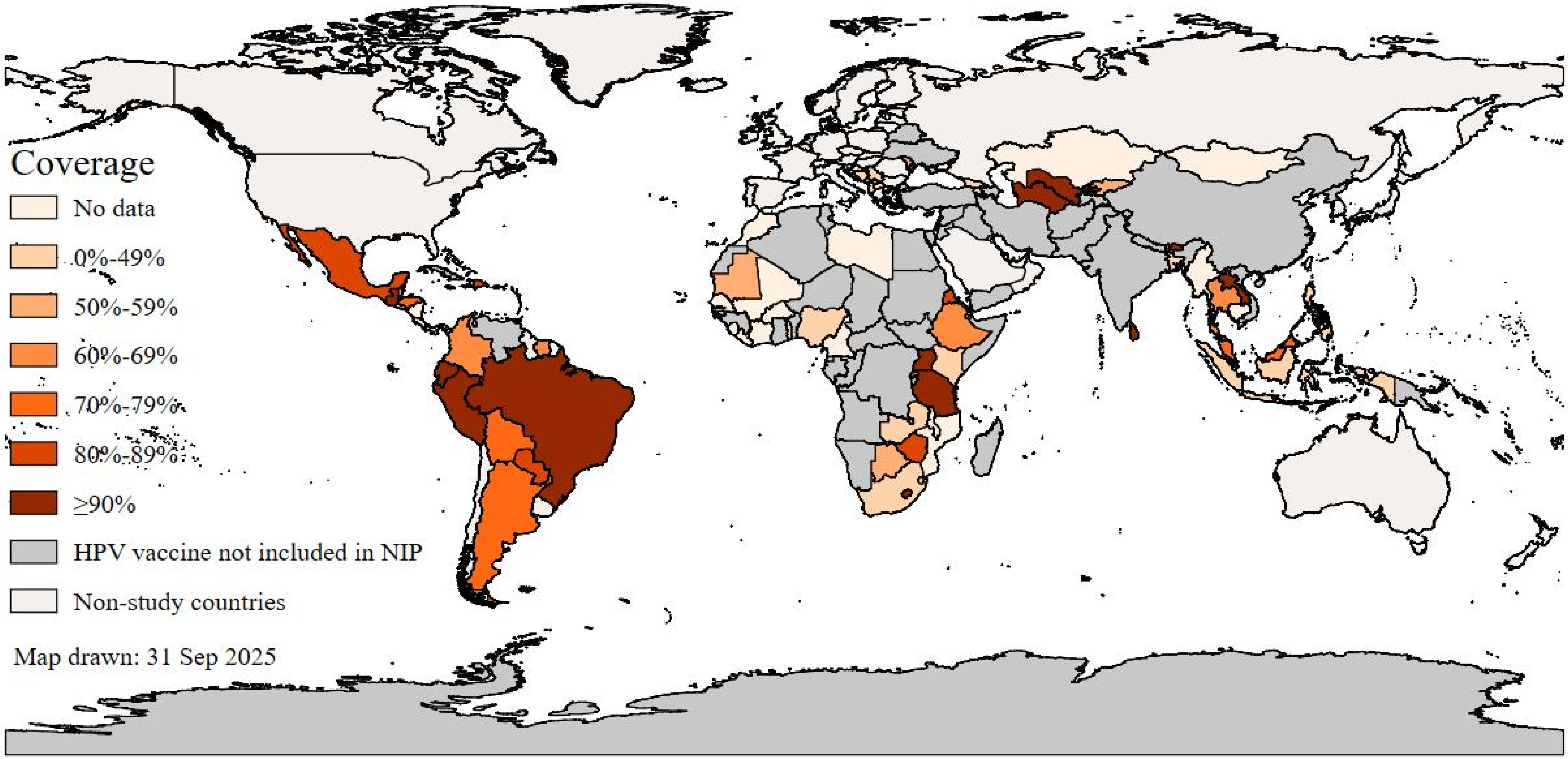

Note: 15HPV1_F: HPV Vaccination coverage by age 15,first dose, females; HPV vaccination coverage in 84 included countries in 2024 was retrieved from WHO website, 62 countries provided 15HPV1_F data.

Supplementary Figure 8. Map of HPV vaccination coverage by age 15, last dose, females in 84 LMICs

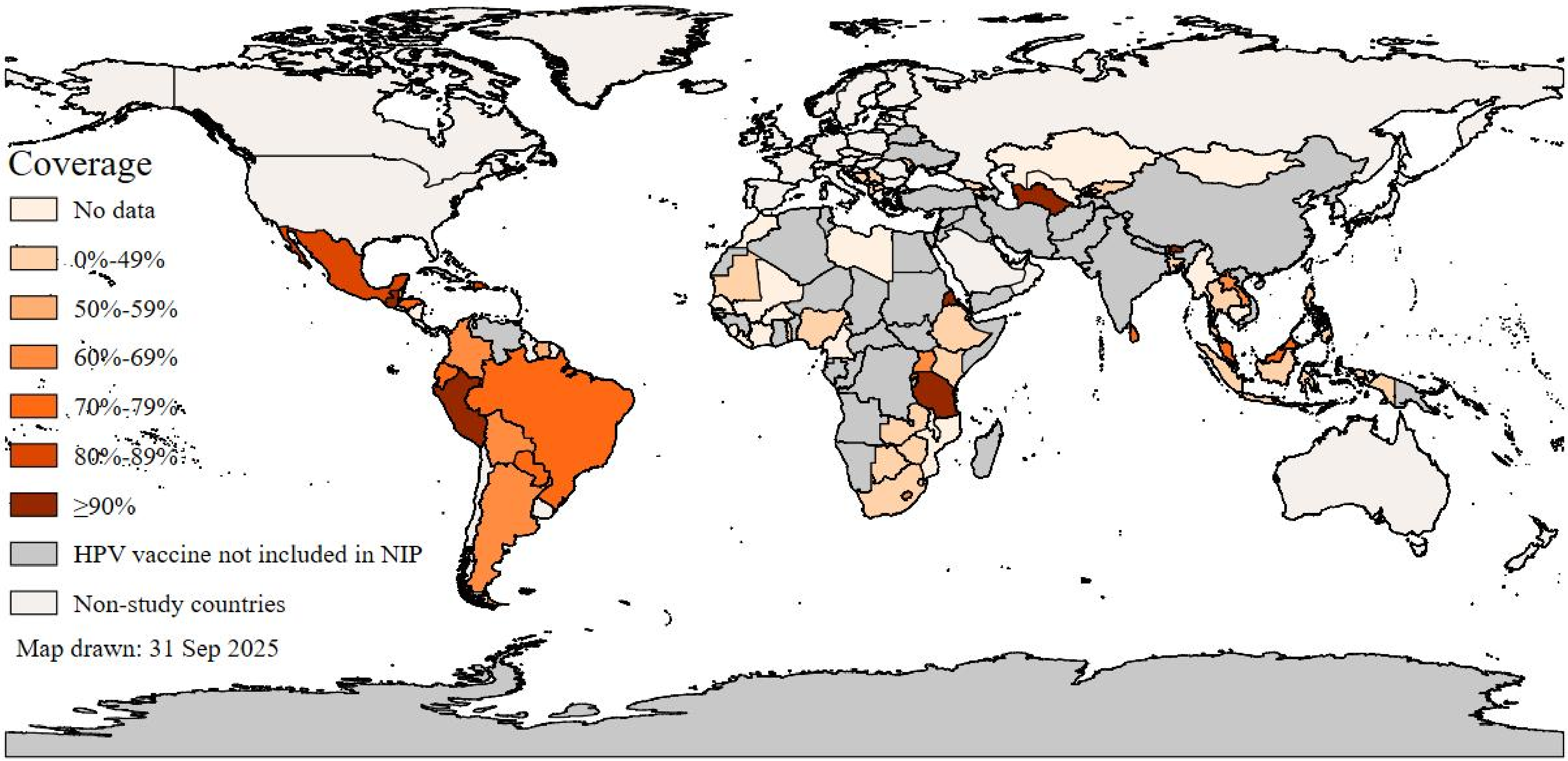

Note: 15HPVC_F: HPV Vaccination coverage by age 15,last dose, females;HPV vaccination coverage in 84 included countries in 2024 was retrieved from WHO website, 61 countries provided 15HPVC_F data.

Supplementary Figure 9. Map of HPV vaccination coverage by age 15, first dose, males in 84 LMICs

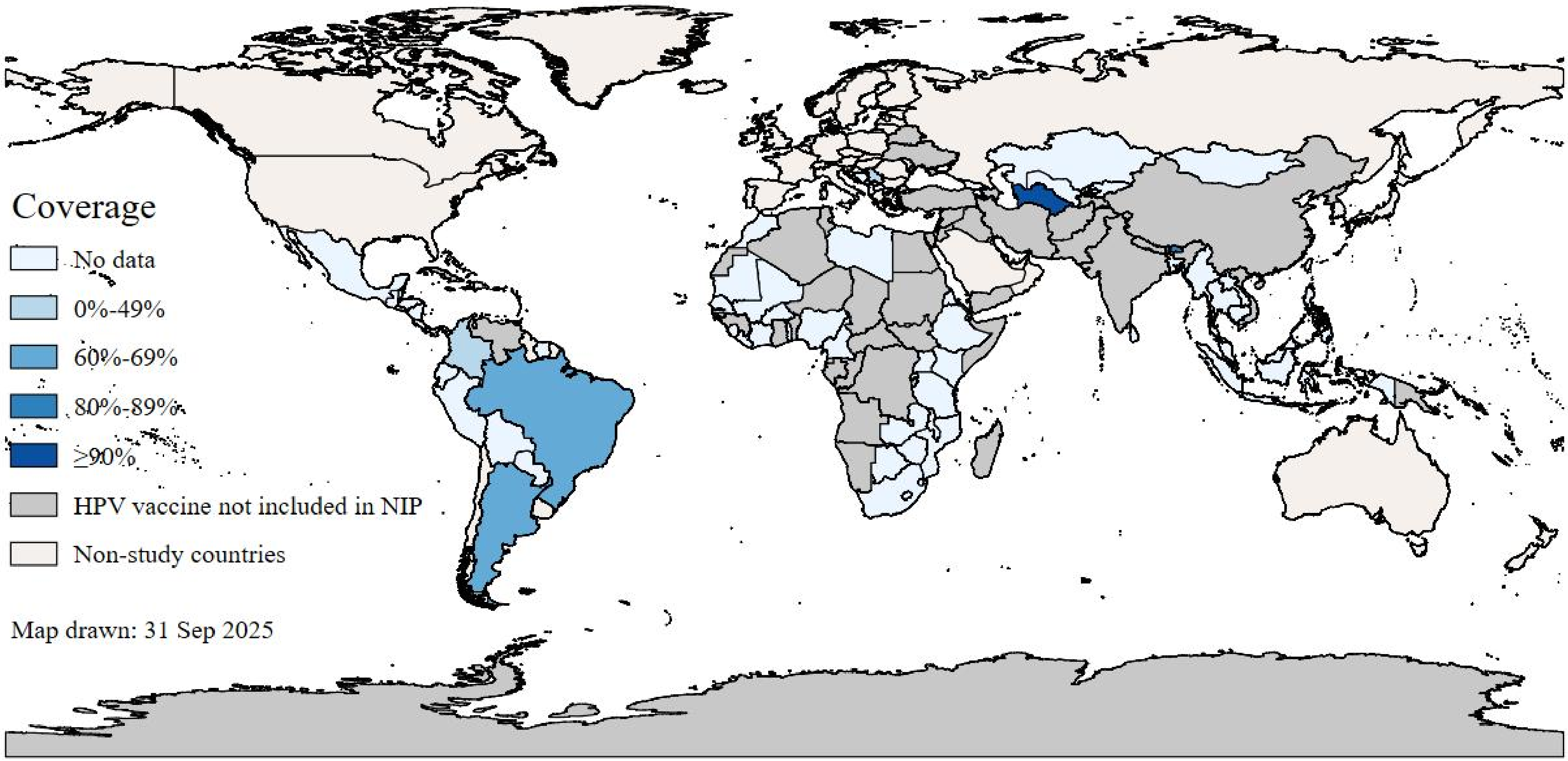

Note: 15HPV1_M: HPV Vaccination coverage by age 15,first dose, males;HPV vaccination coverage in 84 included countries in 2024 was retrieved from WHO website, 10 countries provided 15HPV1_M data.

Supplementary Figure 10. Map of HPV vaccination coverage by age 15, last dose, males in 84 LMICs

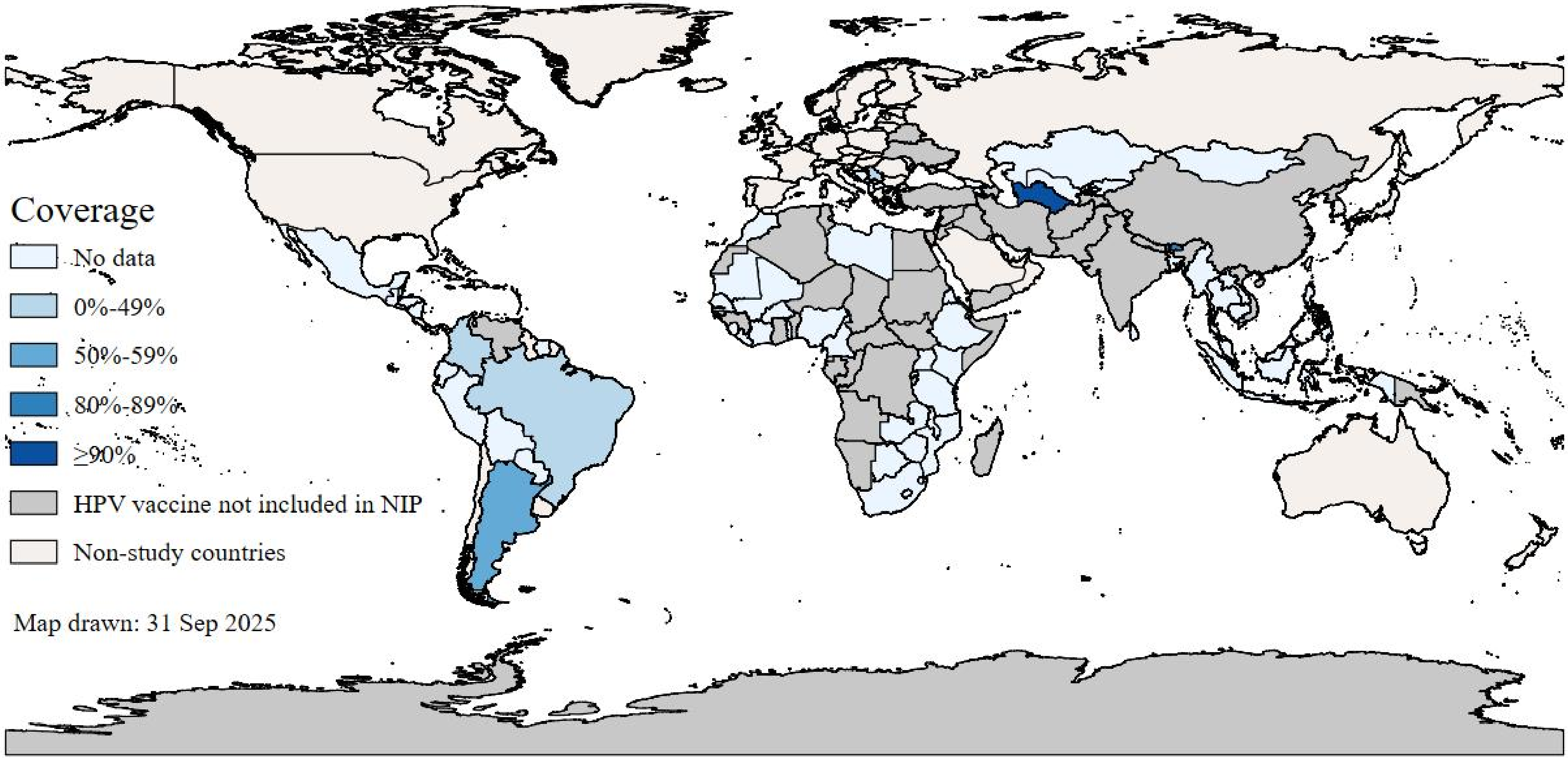

Note: 15HPVC_M: HPV Vaccination coverage by age 15,last dose, males; HPV vaccination coverage in 84 included countries in 2024 was retrieved from WHO website,10 countries provided 15HPVC_M data

Supplementary Figure 11. Trends in HPV Vaccination Coverage by Age 15, Grouped by Dose Regimen (2011-2024)

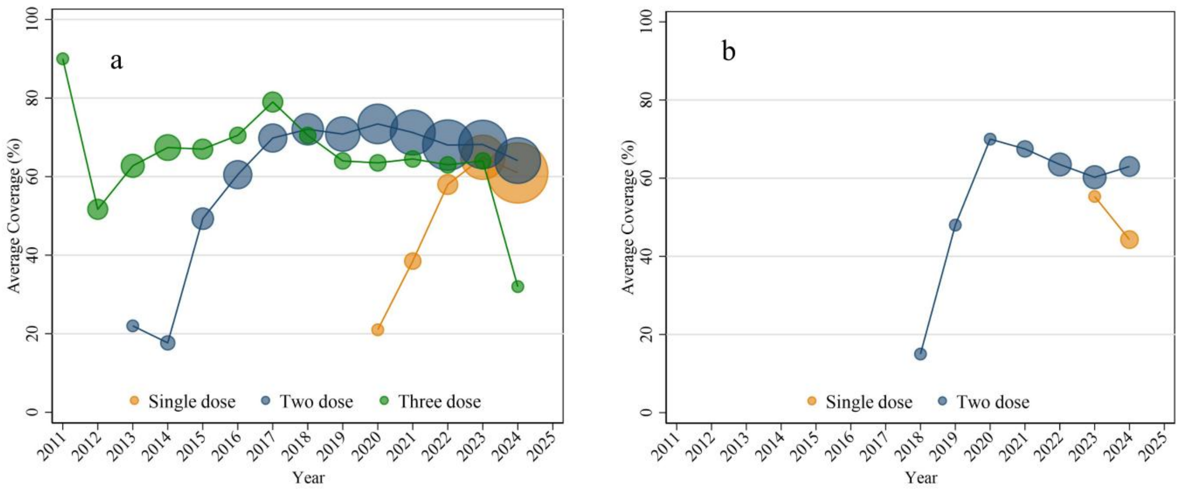

Notes: Supplementary Figure 11a-11b present trends in HPV vaccination coverage by age 15 across 2011-2024, grouped by vaccination dose regimen, corresponding to 15HPV1_F and 15HPV1_M, respectively. Circle size=Number of valid countries (group/year).15HPV1_F: HPV Vaccination coverage by age 15,first dose, females; 15HPV1_M: HPV Vaccination coverage by age 15,first dose, males; HPV vaccination coverage in 84 included countries in 2024 was retrieved from WHO website, 62 countries provided 15HPV1_F data, 10 countries provided 15HPV1_M data. Since several countries changed their schedule during the study period, each country was assigned to its current regimen group on a calendar-year basis, so that its coverage contributes to the post-switch group from the year the change took effect.

Supplementary Figure 12. Trends in HPV Vaccination Coverage by Age 15, Grouped by Rollout Mode (2011-2024)

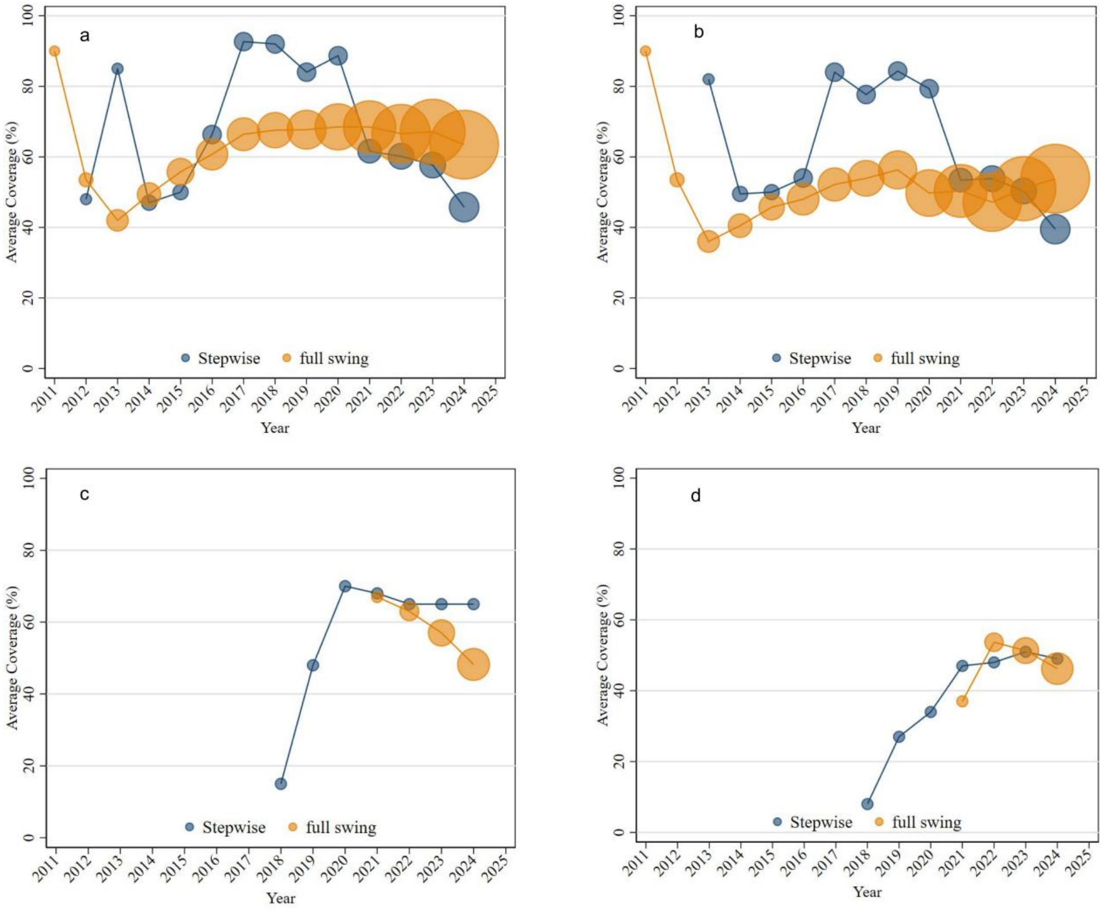

Notes: Supplementary Figure 11a-11d present trends in HPV vaccination coverage by age 15 across 2011-2024, grouped by vaccination rollout mode, corresponding to 15HPV1_F, 15HPVC_F, 15HPV1_M and 15HPVC_M, respectively. Circle size=Number of valid countries (group/year).15HPV1_F: HPV Vaccination coverage by age 15,first dose, females; 15HPVC_F: HPV Vaccination coverage by age 15,last dose, females;15HPV1_M: HPV Vaccination coverage by age 15,first dose, males; 15HPVC_M: HPV Vaccination coverage by age 15,last dose, males; HPV vaccination coverage in 84 included countries in 2024 was retrieved from WHO website, 62 countries provided 15HPV1_F data, 61 countries provided 15HPVC_F data, 10 countries provided 15HPV1_M data and 15HPVC_M data.

Supplementary Table 1. Preferred Reporting Items for Systematic reviews and Meta-Analyses extension for Scoping Reviews (PRISMA-ScR) Checklist

SECTION	ITEM	PRISMA-ScR CHECKLIST ITEM	REPORTED ON PAGE #
**TITLE**			
Title	1	Identify the report as a scoping review.	1
**ABSTRACT**			
Structured summary	2	Provide a structured summary that includes (as applicable): background, objectives, eligibility criteria, sources of evidence, charting methods, results, and conclusions that relate to the review questions and objectives.	2-3
**INTRODUCTION**			
Rationale	3	Describe the rationale for the review in the context of what is already known. Explain why the review questions/objectives lend themselves to a scoping review approach.	5
Objectives	4	Provide an explicit statement of the questions and objectives being addressed with reference to their key elements (e.g., population or participants, concepts, and context) or other relevant key elements used to conceptualize the review questions and/or objectives.	6
**METHODS**			
Protocol and registration	5	Indicate whether a review protocol exists; state if and where it can be accessed (e.g., a Web address); and if available, provide registration information, including the registration number.	N/A
Eligibility criteria	6	Specify characteristics of the sources of evidence used as eligibility criteria (e.g., years considered, language, and publication status), and provide a rationale.	7
Information sources*	7	Describe all information sources in the search (e.g., databases with dates of coverage and contact with authors to identify additional sources), as well as the date the most recent search was executed.	6-7
Search	8	Present the full electronic search strategy for at least 1 database, including any limits used, such that it could be repeated.	6-7
Selection of sources of evidence†	9	State the process for selecting sources of evidence (i.e., screening and eligibility) included in the scoping review.	8
Data charting process‡	10	Describe the methods of charting data from the included sources of evidence (e.g., calibrated forms	8-9

SECTION	ITEM	PRISMA-ScR CHECKLIST ITEM	REPORTED ON PAGE #
		or forms that have been tested by the team before their use, and whether data charting was done independently or in duplicate) and any processes for obtaining and confirming data from investigators.	
Data items	11	List and define all variables for which data were sought and any assumptions and simplifications made.	8-9
Critical appraisal of individual sources of evidence§	12	If done, provide a rationale for conducting a critical appraisal of included sources of evidence; describe the methods used and how this information was used in any data synthesis (if appropriate).	N/A
Synthesis of results	13	Describe the methods of handling and summarizing the data that were charted.	8-9
**RESULTS**			
Selection of sources of evidence	14	Give numbers of sources of evidence screened, assessed for eligibility, and included in the review, with reasons for exclusions at each stage, ideally using a flow diagram.	9
Characteristics of sources of evidence	15	For each source of evidence, present characteristics for which data were charted and provide the citations.	9-10, 14-16
Critical appraisal within sources of evidence	16	If done, present data on critical appraisal of included sources of evidence (see item 12).	N/A
Results of individual sources of evidence	17	For each included source of evidence, present the relevant data that were charted that relate to the review questions and objectives.	9-10, 14-16
Synthesis of results	18	Summarize and/or present the charting results as they relate to the review questions and objectives.	10-16
**DISCUSSION**			
Summary of evidence	19	Summarize the main results (including an overview of concepts, themes, and types of evidence available), link to the review questions and objectives, and consider the relevance to key groups.	16-21
Limitations	20	Discuss the limitations of the scoping review process.	22-23
Conclusions	21	Provide a general interpretation of the results with respect to the review questions and objectives, as well as potential implications and/or next steps.	23
**FUNDING**			
Funding	22	Describe sources of funding for the included	24

SECTION	ITEM	PRISMA-ScR CHECKLIST ITEM	REPORTED ON PAGE #
		sources of evidence, as well as sources of funding for the scoping review. Describe the role of the funders of the scoping review.	

JBI = Joanna Briggs Institute; PRISMA-ScR = Preferred Reporting Items for Systematic reviews and Meta-Analyses extension for Scoping Reviews.

* Where sources of evidence (see second footnote) are compiled from, such as bibliographic databases, social media platforms, and Web sites.

† A more inclusive/heterogeneous term used to account for the different types of evidence or data sources (e.g., quantitative and/or qualitative research, expert opinion, and policy documents) that may be eligible in a scoping review as opposed to only studies. This is not to be confused with information sources (see first footnote).

‡ The frameworks by Arksey and O'Malley (6) and Levac and colleagues (7) and the JBI guidance (4, 5) refer to the process of data extraction in a scoping review as data charting.

§ The process of systematically examining research evidence to assess its validity, results, and relevance before using it to inform a decision. This term is used for items 12 and 19 instead of "risk of bias" (which is more applicable to systematic reviews of interventions) to include and acknowledge the various sources of evidence that may be used in a scoping review (e.g., quantitative and/or qualitative research, expert opinion, and policy document).

From: Tricco AC, Lillie E, Zarin W, O'Brien KK, Colquhoun H, Levac D, et al. PRISMA Extension for Scoping Reviews (PRISMAScR): Checklist and Explanation. Ann Intern Med. 2018;169:467–473. doi: 10.7326/M18-0850.

Supplementary Table 2. Search Strategy Across Different Databases

DATABASE	SEARCH TERM
PubMed	(("implement*"[All Fields] OR "monitor*"[All Fields] OR "cooperation"[All Fields] OR "evaluation"[All Fields] OR "stakeholder"[All Fields] OR "hesitancy"[All Fields] OR "equity"[All Fields] OR ("coverage rate"[All Fields] OR "infection rate"[All Fields] OR "adverse event"[All Fields] OR "antibody level"[All Fields])) AND ("HPV vaccination"[Title/Abstract] OR "HPV immunization"[Title/Abstract] OR "cervical cancer prevention"[Title/Abstract])) AND (1000/1/1:2025/7/31[pdat])
Embase	#1 'HPV vaccination':ti.ab.kw OR 'hpv immunization':ti.ab.kw OR 'cervical cancer prevention':ti.ab.kw<br>#2 'implement*' OR 'monitor* 'OR 'cooperation' OR 'evaluation' OR 'stakeholder' OR 'hesitancy' OR 'equity'<br>#3'coverage rate' OR 'infection rate' OR 'adverse event'OR 'antibody level'<br>#4 #2 OR #3<br>#5 #1 AND #4<br>#6 #1 AND #4 AND [24-08-1000]/sd NOT [01-08-2025]/sd
Cochrane Database of Systematic Review	#1 "HPV vaccination":ti,ab,kw OR "HPV immunization":ti,ab,kw OR "cervical cancer prevention":ti,ab,kw (Word variations have been searched)<br>#2 "implement*" OR "monitor*" OR "cooperation" OR "evaluation" OR "stakeholder" OR "hesitancy" OR "equity" OR "coverage rate" OR "infection rate" OR "adverse event" OR "antibody level" (Word variations have been searched)<br>#3 #1 AND #2
Web of Science	#1 ALL=("coverage rate" OR "infection rate" OR "adverse event" OR "antibody level")<br>#2 TS=("HPV vaccination" or "HPV immunization" or "cervical cancer prevention")<br>#3 ALL=("implement*" or "monitor*" or "cooperation" or "evaluation" or "stakeholder" or "hesitancy" or "equity")<br>#4 #1 OR #3<br>#5 #2 AND #4

Supplementary Table 3. Evolution process of adolescent HPV vaccination strategies in 84 LMICs

Income level	Country （region）	Introduction year	Last update year	History	Key Changes	Age(year)/target population	Sex	Doses	Vaccine type	Delivery platform	Payment strategy	Funding source	Source	Policy Link
Upper-MICs	Albania	2022	NA			13	F	1 dose	Gardasil (4v)	Health facility-based	free	GF and EF	WHO/official web/pubmed	1.https://albania.un.org/en/217753-unfpa-human-papilloma-virus-vaccine-now-available-public-health-centers<br>2.https://shendetesia.gov.al/?s=HPV<br>3.https://www.ncbi.nlm.nih.gov/pmc/articles/PMC10909086/
	Argentina	2011	2024	current		11-20	F and M	1 dose	Gardasil (4v)	Health facility-based	free	GF	official web/pubmed	1.https://europepmc.org/article/MED/26858384<br>2.https://www.argentina.gob.ar/(法案 **7)**<br>**3.https://www.argentina.gob.ar/sites/default/files/2018/02/lineamiento_vph_unica_dosis_2023_1572024.pdf**<br>**4.https://www.hpvworld.com/articles/early-impact-of-hpv-vaccination-in-argentina-strong-reduction-in-prevalence-of-hpv16-18-and-closely-related-hpv-types-in-sexually-active-vaccinated-adolescent-girls/**<br>**5.https://www.boletinoficial.gob.ar/detalleAviso/primera/300983/20231218**
						11-26 ICP	F and M	3 doses (0,2,6 m)	Gardasil (4v)					
				2011	Introduction	11	F	3 doses (0,2,6 m)	Cervarix (2v)					
				2014	Vaccine type change	11	F	3 doses (0,2,6 m)	Gardasil (4v)					
						11-26 ICP	F	3 doses (0,2,6 m)	Gardasil (4v)					
				2015	Dose reduction	11	F	2 doses (0,6 m)	Gardasil (4v)					
						11-26 ICP	F	3 doses (0,2,6 m)	Gardasil (4v)					
				2017	Expanded to boys	11	F and M	2 doses (0,6 m)	Gardasil (4v)					
						11-26 ICP	F and M	2 doses (0,6 m)	Gardasil (4v)					
				2024	Further dose reduction	11-20	F and M	1 dose	Gardasil (4v)					
						11-26 ICP	F and M	3 doses (0,2,6 m)	Gardasil (4v)					
	Armenia	2017	2019	current		13-45	F	2 doses (0,6 m)	Gardasil (4v)	Health facility-based	free	GF and EF	pubmed	1.https://www.ncbi.nlm.nih.gov/pmc/articles/PMC10909086/<br>2.https://www.ncbi.nlm.nih.gov/pmc/articles/PMC10495901/
						14-45	M	2 doses (0,6 m)	Gardasil (4v)					
				2017	Introduction	13	F	2 doses (0,6 m)	Gardasil (4v)					
				2019	Expand age	13-45	F	2 doses (0,6 m)	Gardasil (4v)					

					range									
					Expand age range	14-45	M	2 doses (0,6 m)	Gardasil (4v)					
	Belize	2016	2023	current		9-15	F and M	1 dose	Gardasil (4v)	School based/Health facilities (unschooled)	free	GF	official web/pubmed	1.https://iris.paho.org/bitstream/handle/10665.2/54831/PAHOFPLIM210036_eng.pdf?sequence=1&isAllowed=y<br>2.https://www.pressoffice.gov.bz/ministry-of-health-wellness-advises-on-new-dosage-of-hpv-vaccine/<br>3.https://pubmed.ncbi.nlm.nih.gov/25919158/
				2016	Introduction	9-15	F	2 doses	Gardasil (4v)					
				2018	Expanded to boys	9-15	F and M	2 doses	Gardasil (4v)					
				2023	Dose reduction	9-15	F and M	1 dose	Gardasil (4v)					
	Bosnia and Herzegovina	2022(FBiH. Canton Sarajevo)	NA			11-26	F	2 doses (0,6 m)	Gardasil (4v)	Health facility-based	free	NA	UNFPA/pubmed	1.https://ba.unfpa.org/en/news/road-eradicating-cervical-cancer-bosnia-and-herzegovina<br>2.https://ba.unfpa.org/en/news/hpv-vaccination-programme-implementation-set-begin-sarajevo-canton<br>3.https://ba.unfpa.org/en/news/road-eradicating-cervical-cancer-bosnia-and-herzegovina<br>4.https://ba.unfpa.org/en/publications/comprehensive-cervical-cancer-prevention-and-control-programme-guidance-countries<br>5.https://www.ncbi.nlm.nih.gov/pmc/articles/PMC10909086/
		2023(FBiH. Elsewhere in FBiH)	NA			13-14	F	2 doses (0,6 m)	Gardasil (4v)					
		2023(RS)	NA			11-14	F and M	2 doses (0,6 m)	Gardasil 9 (9v)					
	Botswana	2015	2016	current		9 (Grade 5 in primary school)	F	2 doses (0,6 m)	Gardasil (4v)	School based/Health facilities (unschooled)	free	NA	WHO/official web/pubmed	1.https://www.afro.who.int/regional-director/speeches-messages/hpv-vaccination-africa-game-changer-womens-and-girls-health<br>2.https://www.moh.gov.bw/Publications/frequently_asked_questions/FAQ_hpv_vaccine.pdf<br>3.https://www.ncbi.nlm.nih.gov/pmc/articles/PMC4630577/
				2015	Introduction and catch-up	9-13 (Grades 5-7 in primary school)	F	2 doses (0,6 m)	Gardasil (4v)					
				2016	Routine	9 (Grade 5 in primary school)	F	2 doses (0,6 m)	Gardasil (4v)					
	Brazil	2014	2024	current		9-14	F and M	1 dose	Gardasil (4v)	Health facility-based	free	GF	WHO/official web/pubmed	1.https://www.iarc.who.int/news-events/why-is-brazil-changing-its-hpv-vaccine-recommendations-video-with-dr-iacopo-baussano/
						9-45 ICP	F and M	3 doses (0,2,6 m)	Gardasil (4v)					

				2014	Introduction	11-13	F	2 doses (0,6~12 m)	Gardasil (4v)	School-based				2.https://www.gov.br/saude/pt-br/search?SearchableText=HPV<br>2.https://bmcpublichealth.biomedcentral.com/articles/10.1186/s12889-020-08561-4<br>3.https://www.scirp.org/journal/paperinformation?paperid=123157
				2015	Expand age range	9-13	F	2 doses (0,6~12 m)	Gardasil (4v)	Health facility-based				
				2016	Included ICP	9-26 ICP	F	3 doses (0,2,6 m)	Gardasil (4v)					
				2017	Expanded to boys and Expanded age range	9-14 F and 11-14 M	F and M	2 doses (0,6~12 m)	Gardasil (4v)					
						9-26 ICP	F and M	3 doses (0,2,6 m)	Gardasil (4v)					
				2022	Expand age range	9-14	F and M	2 doses (0,6~12 m)	Gardasil (4v)					
						9-45 ICP	F and M	3 doses (0,2,6 m)	Gardasil (4v)					
				2024	Dose reduction	9-14	F and M	1 dose	Gardasil (4v)					
						9-45 ICP	F and M	3 doses (0,2,6 m)	Gardasil (4v)					
	Colombia	2012	2024	current		9-17	F and M	1 dose	Gardasil (4v)	School-based	free	GF	WHO/UICC/pubmed	1.https://immunizationdata.who.int/global/wiise-detail-page/vaccination-schedule-for-human-papilloma-virus?ISO_3_CODE=COL&TARGETPOP_GENERAL=<br>2.https://www.who.int/news/item/05-03-2024-wave-of-new-commitments-marks-historic-step-towards-the-elimination-of-cervical-cancer<br>3.https://www.uicc.org/case-studies/addressing-stigma-hard-reach-communities-colombia<br>4.https://pmc.ncbi.nlm.nih.gov/articles/PMC9662904/
				2012	Introduction	9-12	F	3 doses (0,2,6 m)	Gardasil (4v)					
				2013	Expand age range	9-17	F	3 doses (0,2,6 m)	Gardasil (4v)					
				2018	Dose reduction	9-17	F	2 doses (0,6 m)	Gardasil (4v)					
				2023	Dose reduction and expanded to boys	9-17 F and 9 M	F and M	1 dose	Gardasil (4v)					
				2024	Expand age range	9-17 F and 9-14 M	F and M	1 dose	Gardasil (4v)					
	Dominica	2019	2023	current		10-12	F and M	1 dose	Gardasil (4v)	School-based	free	GF	WHO	1.https://immunizationdata.who.int/global/wiise-detail-page/vaccinatio

				2019	Introduction	10-12	F and M	2 doses (0,6 m)	Gardasil (4v)					n-schedule-for-human-papilloma-virus?ISO_3_CODE=DMA&TARGETPOP_GENERAL=
				2023	Dose reduction	10-12	F and M	1 dose	Gardasil (4v)					
	Dominican Republic	2017	NA			9-14	F	1 dose	Gardasil (4v)	Health facility-based	free	NA	WHO	https://immunizationdata.who.int/global/wiise-detail-page/vaccination-schedule-for-human-papilloma-virus?ISO_3_CODE=DOM&TARGETPOP_GENERAL=
	Ecuador	2014	NA			9-11	F	2 doses (0,6 m)	Cervarix (2v)	School-based	free	NA	official web/pubmed	1.https://www.salud.gob.ec/wp-content/uploads/2021/10/ESQUEMA-DE-VACUNACIO%CC%81N.oct_.2021.pdf<br>2.https://www.ncbi.nlm.nih.gov/pmc/articles/PMC6660837/<br>3.https://www.shoreland.com/downloads/pdf/ecuador-vaccine-handout_sample.pdf<br>4.https://www.iccp-portal.org/system/files/plans/cervical-cancer-ecu-2021-country-profile-en.pdf
	El Salvador	2020	NA			9	F	2 doses (0,6 m)	Gardasil (4v)	School-based	free	GF and EF	official web/world bank/pubmed	1.https://www.salud.gob.ec/<br>2.https://www.ncbi.nlm.nih.gov/pmc/articles/PMC9179345/<br>3.https://blogs.worldbank.org/en/health/investing-health-women-and-girls-scaling-access-hpv-vaccination
	Fiji	2013	2015	current		8-13	F	2 doses (0,6 m)	Cervarix (2v)	School-based	free	GF and EF	official web/pubmed	1.https://www.health.gov.fj/immunisation/<br>2.https://www.ncbi.nlm.nih.gov/pmc/articles/PMC10285272/<br>3.https://academic.oup.com/cid/article/64/7/852/2747464
				2013	Introduction	8-13	F	3 doses	Cervarix (2v)					
				2015	Dose reduction	8-13	F	2 doses (0,6 m)	Cervarix (2v)					
	Georgia	2019	2023	current		10-46 F and 10-26 M	F and M	2 doses (=<14 years, 0,6 m)；**3 doses (>=15 years,0,2,6 m)**	Gardasil (4v)	Health facility-based	free	GF and EF	WHO/official web/UNFPA/pubmed	1.https://immunizationdata.who.int/global/wiise-detail-page/vaccination-schedule-for-human-papilloma-virus?ISO_3_CODE=GEO&TARGETPOP_GENERAL=<br>2.https://dph.georgia.gov/sites/dph.georgia.gov/files/Immunizations/HPV%2008.25.15.pdf<br>3.https://georgia.unfpa.org/en/news/cervical-cancer-preventable-and-c
				2019	Introduction	10-12	F	2 doses (0,6 m)	Gardasil (4v)					
				2022	catch-up	13-18	F	3 doses (0,2,6 m)	Gardasil (4v)					

				2023	Expanded to boys and Expanded age range	10-46 F and 10-26 M	F and M	2 doses (=<14 years, 0,6 m)；**3 doses (>=15 years,0,2,6 m)**	Gardasil (4v)					urable-1<br>4.https://www.ncbi.nlm.nih.gov/pmc/articles/PMC7957340/
	Grenada	2019	2023	current		9-10	F and M	1 dose	Gardasil (4v)	School-based	free	GF and EF	WHO/official web	1.https://immunizationdata.who.int/global/wiise-detail-page/vaccination-schedule-for-human-papilloma-virus?ISO_3_CODE=GRD&TARGETPOP_GENERAL=<br>2.https://www.cijn.org/hpv-vaccine-a-positive-hope-for-the-future-in-the-battle-against-cervical-cancer/
				2019	Introduction	9-10	F and M	2 doses (0,6 m)	Gardasil (4v)					
				2023	Dose reduction	9-10	F and M	1 dose	Gardasil (4v)					
	Guatemala	2018	2024	current		9-17 F and 9 M	F and M	1 dose	Gardasil (4v)	School-based	free	NA	UICC/pubmed	1.https://hpvcentre.net/statistics/reports/GTM.pdf<br>2.https://www.scielo.org.mx/scielo.php?script=sci_arttext&pid=S0036-36342018000600012<br>3.https://www.ncbi.nlm.nih.gov/pmc/articles/PMC9645205/
				2018	Introduction	9-17	F	1 dose	Gardasil (4v)					
				2024	Expanded to boys	9-17 F and 9 M	F and M	1 dose	Gardasil (4v)					
	Indonesia	2017	NA			11	F	2 doses (0,12 m)	Gardasil (4v)	School-based	free	GF and EF	WHO/official web/IVAC	1.https://www.who.int/indonesia/news/detail/09-08-2023-national-launch-of-human-papillomavirus-(hpv)-immunization-expansion<br>2.https://setkab.go.id/en/health-ministry-expands-free-hpv-vaccination-coverage/<br>3.https://www.iccp-portal.org/system/files/plans/National%20Cervical%20Cancer%20Elimination%20Plan%20for%20Indonesia%202023-2030_compressed.pdf<br>4.https://publichealth.jhu.edu/sites/default/files/2024-02/3advocacy-needs-for-hpv-vaccines-in-indonesiaax.pdf<br>5.https://www.gavi.org/vaccineswork/gavi-impact-asia-and-pacific-2000?f_link_type=f_linkinlinenote&flow_extra=eyJpbmxpbmVfZGlzcGxheV9wb3NpdGlvbiI6MCwiZG9jX3Bvc2l0aW9uIjowLCJkb2NfaWQiOiJkYzBlZWEyNThiMGNjMmZlLTM5NGI0MDc5Mzk2ZDNjYj

														EifQ%3D%3D
	Jamaica	2017	NA			9-14	F and M	1 dose	NA	School-based	free	NA	official web	1.https://www.moh.gov.jm/edu-resources/human-papillomavirus-hpv-vaccine/ 2.https://vaccination.moh.gov.jm/hpv-vaccine/ 3.https://jis.gov.jm/information/get-the-facts/get-the-facts-human-papillomavirus-hpv-vaccination-programme/ 4.https://www.google.com/url?sa=t&rct=j&q=&esrc=s&source=web&cd=&ved=2ahUKEwi_yMyN9JmHAxVasVYBHSN-ChoQFnoECA4QAw&url=https%3A%2F%2Fwww.paho.org%2Fen%2Fnews%2F25-4-2024-jamaica-launches-22nd-vaccination-week-americas-promote-immunization&usg=AOvVaw1n4yXqlg2Q67WErVw9oe_p&opi=89978449
						15-26	F	2 doses (0,6 m)	NA					
						ICP	F and M	3 doses (0,2,6 m)	NA					
	Kazakhstan	2024	2024	current		11	F	2 doses	Gardasil (4v)	NA	free	NA	official news web	1.https://new.kaztag.kz/en/news/over-278-000-adolescents-vaccinated-against-hpv-in-kazakhstan； **2.https://www.who.int/europe/news/item/19-01-2024-protecting-a-generation--kazakhstan-s-commitment-to-hpv-vaccination；** **3.https://qazinform.com/news/kazakhstan-may-vaccinate-boys-ag**

														**ainst-human-papillomavirus-6d7321**
	Libya	2013	2017	current		12	F	3 doses (0,1,6 m)	Gardasil (4v)	NA	free	GF and EF	WHO/official web	1.https://immunizationdata.who.int/global/wiise-detail-page/vaccination-schedule-for-human-papilloma-virus?ISO_3_CODE=LBY&TARGETPOP_GENERAL=<br>2.https://www.who.int/about/accountability/results/who-results-report-2020-mtr/country-story/2021/liberia<br>3.https://www.fondation-merieux.org/wp-content/uploads/2017/01/vaccine-benefits-2017-suleiman-abusrewil.pdf
				2013	Introduction	15	F	3 doses (0,1,6 m)	Gardasil (4v)					
				2017	Moved age	12	F	3 doses (0,1,6 m)	Gardasil (4v)					
					Catch-up	13-14	F	3 doses (0,1,6 m)	Gardasil (4v)					
	Malaysia	2010	2017	current		13	F	2 doses (0,6 m)	Cervarix (2v), Gardasil (4v)	School-based	free	GF	official web/UICC/Pubmed	1.https://hpv.cancer.org.my/en/leaving-no-one-behind<br>2.http://www.myhealth.gov.my/en/vaccine-hpv/<br>3.https://adultimmunisation.msidc.my/human-papillomavirus/<br>4.https://www.uicc.org/case-studies/harnessing-media-platforms-hpv-advocacy-malaysia<br>5.https://www.cancercontrol.info/2020-4/lowering-the-burden-of-cancer-in-a-middle-income-country-the-idealist-versus-the-pragmatist-approach/<br>6.https://journals.plos.org/plosone/article?id=10.1371/journal.pone.0278477<br>7.https://www.assunta.com.my/cervical-cancer-an-evolving-solution/
				2010	Introduction	13	F	3 doses (0,1,6 m)	Cervarix (2v), Gardasil (4v)					
				2013	Catch-up	16-21	F	3 doses (0,1,6 m)	Cervarix (2v), Gardasil (4v)					
				2015	Dose reduction	13	F	2 doses (0,6 m)	Cervarix (2v), Gardasil (4v)					
				2017	Vaccine type change	13	F	2 doses (0,6 m)	Gardasil (9v)					
	Maldives	2019	NA			10-14	F	2 doses (0,6 m)	NA	School-based	free	GF and EF	WHO	1.https://www.who.int/about/accountability/results/who-results-report-

														2020-mtr/country-story/2021/maldives<br>2.https://health.gov.mv/storage/uploads/NQoQXdqv/iazle9aq.pdf<br>3.https://maldives.un.org/en/175650-who-provides-support-maldives-conducting-joint-covid-19-and-hpv-vaccines-post-introduction<br>4.https://www.ncbi.nlm.nih.gov/pmc/articles/PMC7957340/
	Marshall Islands	2009	NA			11-12	F	2 doses (0,6 m)	NA	School-based	free	GF	official web/pubmed	1.https://mchb.tvisdata.hrsa.gov/Narratives/PlanForTheApplicationYear4/6504e31c-a572-48d1-91b6-06796115b04c<br>2.https://www.ncbi.nlm.nih.gov/pmc/articles/PMC7957340/
	Mauritius	2016	2023	current		9-15	F and M	2 doses (0,6 m)	Cervarix (2v)	School-based	free	GF	WHO	1.https://www.afro.who.int/countries/mauritius/news/mauritius-moving-step-ahead-its-fight-against-cancer-extending-vaccination-against-human<br>2.https://www.ncbi.nlm.nih.gov/pmc/articles/PMC7957340/<br>3.https://hpvcentre.net/statistics/reports/MUS.pdf<br>4.https://health.govmu.org/health/wp-content/uploads/2023/03/National-Cancer-Control-Programme-2022-2025.pdf
				2016	Introduction	9	F	2 doses (0,6 m)	Cervarix (2v)					
				2023	Expanded to boys and Expanded age range	9-15	F and M	2 doses (0,6 m)	Cervarix (2v)					
	Mexico	2012	2023	current		11-13	F	1 dose	Gardasil (4v)	School-based/health facilities (unschooled)	free	GF	WHO/IVAC/pubmed/official news web	1.https://immunizationdata.who.int/global/wiise-detail-page/human-papillomavirus-(hpv)-vaccination-coverage?CODE=MEX&YEAR=<br>2.https://www.hpvworld.com/articles/mexico-human-papillomavirus-and-related-cancers-fact-sheet-2019/<br>3.https://hpvcentre.net/statistics/reports/MEX.pdf<br>4.https://www.cureus.com/articles/253043-cervical-cancer-in-mexico-from-a-renowned-vaccination-program-to-unfulfilled-needs-in-treatment-access#!/<br>5.https://theyucatantimes.com/2023/10/imss-announces-human-papilloma-virus-vaccination-campaign-for-11-12-and-13-year-old-girls/
						11-49	F (with HIV)	3 doses (0,1,6 m)	Gardasil (4v)					
				2012	Introduction	11-13	F	3 doses	Gardasil (4v)					
				2014	Dose change and Included women aged 11 to 49 living with HIV	11-13	F	2 doses (0,6 m)	Gardasil (4v)					
						11-49	F (with HIV)	3 doses (0,1,6 m)	Gardasil (4v)					

				2023	Dose change	11-13	F	1 dose	Gardasil (4v)					
						11-49	F (with HIV)	3 doses (0,1,6 m)	Gardasil (4v)					
	Montenegro	2022	2023	current		9-14	F	1 dose	Gardasil 9 (9v)	Health facility-based	free	NA	WHO/pubmed	1.https://immunizationdata.who.int/global/wiise-detail-page/vaccination-schedule-for-human-papilloma-virus?ISO_3_CODE=MEX&TARGETPOP_GENERAL= 2.https://www.who.int/about/accountability/results/who-results-report-2020-mtr/country-story/2022/introducing-hpv-vaccines-for-a-cervical-cancer-free-generation-in-montenegro 3.https://www.who.int/about/accountability/results/who-results-report-2022-mtr/country-story/2022/introducing-hpv-vaccines-for-a-cervical-cancer-free-generation-in-montenegro 4.https://pubmed.ncbi.nlm.nih.gov/39066441/
				2022	Introduction	9	F	1 dose	Gardasil 9 (9v)					
				2023	Expanded age range	9-14	F	1 dose	Gardasil 9 (9v)					
	North Macedonia	2009	2024	current		12-19	F and M	3 doses	Gardasil (4v)	School-based	free	GF	WHO/pubmed	1.https://zdravstvo.gov.mk/wp-content/uploads/2013/01/%3f%3f%3f%3f%3f%3f%3f-hpv.pdf 2.https://www.iph.mk/mk/news/index/78 3.https://eeca.unfpa.org/en/news/north-macedonia-expands-free-hpv-vaccination-programme-adolescent-girls-and-boys
				2009	Introduction	12	F	3 doses (0,2,6 m)	Gardasil (4v)					
				2024	Expanded to boys, Expanded age range, and vaccine type changed	12-19	F and M	3 doses (0,2,6 m)	Gardasil 9 (9v)					
	Paraguay	2013	2024	current		9-14	F and M	1 dose	Gardasil (4v)	School-based	free	GF	official web/pubmed	1.https://pai.mspbs.gov.py/?s=vacuna+VPH 2.https://pubmed.ncbi.nlm.nih.gov/37023094/
				2013	Introduction	9-10	F	3 doses (0,1,6 m)	Gardasil (4v)					
				2024	Dose reduction and Expanded to boys	9-14	F and M	1 dose	Gardasil (4v)					
	Peru	2015	2024	current		9-18	F and M	1 dose	Gardasil (4v)	School-based	free	GF and EF	official	1.https://www.gov.br/saude/pt-br/centrais-de-conteudo/publicacoes/no

				2015	Introduction	9-13	F	2 doses (0,6 m)	Gardasil (4v)				web/other publishing platform/official news web	tas-tecnicas/2024/nota-tecnica-no-41-2024-cgici-dpni-svsa-ms<br>2.http://www.scielo.org.pe/scielo.php?pid=S2304-51322020000400006&script=sci_arttext&tlng=en<br>3.https://diariocorreo.pe/peru/minsa-extiende-la-vacunacion-contra-el-virus-del-papiloma-humano-hasta-los-18-anos-noticia/<br>4.https://www.52hrtt.com/ru/n/w/info/A1752028082798
				2023	Dose reduction	9-13	F and M	1 dose	Gardasil (4v)					
				2024	Expand age range	9-18	F and M	1 dose	Gardasil (4v)					
	Republic of Moldova	2017	2021	current		9-14	F and M	2 doses (0,6 m)	Gardasil (4v)	Health facility-based	free	GF	Gavi/pubmed	1.https://demandhub.org/wp-content/uploads/2024/06/Demand-Hub_HPV-Technical-Session-2-29-May-2024.pdf<br>2.https://journal.waocp.org/?sid=Entrez:PubMed&id=pmid:38156858&key=2023.24.12.4227
				2017	Introduction(partial)	10	F	2 doses (0,6 m)	Gardasil (4v)			EF		
				2021	Expanded to boys and Increased age range	9-14	F and M	2 doses (0,6 m)	Gardasil (4v)			GF		
	Saint Lucia	2019	2025	current		11-12	F and M	1 dose	Gardasil 9 (9v)	School-based	free	GF	official web	1.https://socialtransformation.govt.lc/news/department-of-health-approves-hpv-vaccine-for-students。<br>2.https://www.govt.lc/news/countries-of-the-americas-to-have-access-to-the-hpv9-vaccine?utm_source=chatgpt.com
				2019	Introduction	11-12	F and M	2 doses	Gardasil (4v)					
				2023	Dose reduction	11-12	F and M	1 dose	Gardasil (4v)					
				2025	Vaccine type change	11-12	F and M	1 dose	Gardasil 9 (9v)					
	Saint Vincent and the Grenadines	2017	NA			11-12	F	NA	Cervarix (2v)	School-based	free	GF and EF	WHO	1.https://immunizationdata.who.int/global/wiise-detail-page/vaccination-schedule-for-human-papilloma-virus?ISO_3_CODE=VCT&TARGETPOP_GENERAL=<br>2.https://www.who.int/publications/m/item/cervical-cancer-vct-country-profile-2021
	Serbia	2022	NA			9-14	F and M	2 doses (0,6 m)	Gardasil 9 (9v)	Health facility-based	free	SHI	official web	1.https://www.batut.org.rs/index.php?content=2383<br>2.https://www.batut.org.rs/download/aktuelno/HPV%20Pitanja%20i%

						15-19	F and M	3 doses (0,2,6 m)	Gardasil 9 (9v)					20odgovori%20logo.pdf
	South Africa	2014	2024	current		≥9 in Grade 4 in primary school	F	1 dose	Cervarix (2v)	School-based	free	GF	official web	1.South Africa: HPV Vaccination Switching to Single-Dose and Private Schools to Get Government Supply - allAfrica.com 2.https://www.ghspjournal.org/content/ghsp/6/3/425.full.pdf 3.https://www.nicd.ac.za/wp-content/uploads/2017/08/NICD_Vaccine_Booklet_D132_FINAL.pdf 4.https://allafrica.com/stories/202406190527.html 5.https://www.health.gov.za/wp-content/uploads/2021/07/cervical-cancer-policy.pdf
				2014	Introduction	≥9 in Grade 4 in primary school	F	2 doses (0,6 m)	Cervarix (2v)					
				2024	Dose reduction	≥9 in Grade 4 in primary school	F	1 dose	Cervarix (2v)					
	Suriname	2013	2023	current		9-13	F and M	2 doses (0,6 m)	Gardasil (4v)	School-based	NA	NA	WHO/official web	1.https://immunizationdata.who.int/global/wiise-detail-page/vaccination-schedule-for-country_name?DISEASECODE=&TARGETPOP_GENERAL= 2.https://immunizationdata.who.int/global/wiise-detail-page/human-papillomavirus-(hpv)-vaccination-coverage?CODE=SUR&ANTIGEN=&YEAR= 3.https://www.paho.org/en/news/29-11-2023-paho-suriname-gears-fight-against-cervical-cancer-hpv-vaccine-training
				2013	Introduction	9-13	F	2 doses (0,6 m)	Gardasil (4v)					
				2023	Extension to boys	9-13	F and M	2 doses (0,6 m)	Gardasil (4v)					
	Thailand	2017	NA			11 (Grade 5 in primary school)	F	2 doses (0,6 m)	Gardasil (4v)	School-based	free	NA	IVAC	https://hpvcentre.net/statistics/reports/THA.pdf
	Tonga	2022	2023	current		10	F	1 dose	Cervarix (2v)	School-based/Health facilities	free	NA	WHO/pubmed	1.https://immunizationdata.who.int/global/wiise-detail-page/vaccination-schedule-for-country_name?DISEASECODE=&TARGETPOP_GENERAL= 2.https://pubmed.ncbi.nlm.nih.gov/40412335/
				2022	Introduction and catch-up	10-17	F	2 doses (0,6 m)	Cervarix (2v)					
				2023	Dose reduction and routine	10	F	1 dose	Cervarix (2v)					
				2023	Catch-up	10-14	F	1 dose	Cervarix (2v)					

	Turkmenistan	2016	NA			9	F and M	2 doses (0,6 m)	Gardasil (4v)	Mixed (schools and health facilities)	free	GF and EF	WHO/pubmed	1.https://www.who.int/azerbaijan/news/item/04-07-2024-first-qualitative-research-study-conducted-in-turkmenistan-focuses-on-hpv-vaccination<br>2.https://pubmed.ncbi.nlm.nih.gov/38156858/
	Tuvalu	2021	NA			10	F	1 dose	Cervarix (2v)	NA	free	NA	WHO	1.https://immunizationdata.who.int/global/wiise-detail-page/vaccination-schedule-for-human-papilloma-virus?ISO_3_CODE=TUV&TARGETPOP_GENERAL=
Lower-MICs	Bangladesh	2023	NA			10-14	F	1 dose	Cervarix (2v)	Mixed (schools and health facilities for unschooled)	free	EF	WHO	1.https://www.who.int/bangladesh/news/detail/10-10-2023-human-papillomavirus-(hpv)-vaccination-launching-in-bangladesh--a-single-dose-vaccine-has-potential-to-prevent-cervical-cancer
	Bhutan	2010	2021	current		12	F and M	2 doses (0,6 m)	Gardasil (4v)	School-based	free	GF	WHO/official web/Gavi/pubmed	1.https://immunizationdata.who.int/global/wiise-detail-page/vaccination-schedule-for-human-papilloma-virus?ISO_3_CODE=BTN&TARGETPOP_GENERAL=<br>2.https://www.iarc.who.int/news-events/towards-cervical-cancer-elimination-through-hpv-vaccination-in-bhutan/<br>3.https://www.moh.gov.bt/re-schedule-for-school-hpv-vaccination-for-2015-session-only/<br>4.https://www.gavi.org/news/media-room/government-bangladesh-launches-nationwide-human-papillomavirus-hpv-vaccination<br>5.https://www.ncbi.nlm.nih.gov/pmc/articles/PMC9970746/<br>6.https://www.sciencedirect.com/science/article/abs/pii/S0264410X15007513<br>7.https://obgyn.onlinelibrary.wiley.com/doi/full/10.1002/ijgo.13728<br>8.https://www.sci-hub.st/10.1016/j.vaccine.2015.05.078
				2010	Introduction and catch-up	12-18	F	3 doses (0,2,6 m)	Gardasil (4v)	School-based				
				2011	Routine	12	F	3 doses (0,2,6 m)	Gardasil (4v)	Health facility-based				
				2014	Routine	Grade 6 in primary school	F	3 doses (0,2,6 m)	Gardasil (4v)	School-based				
				2016	Routine	Grade 6 in primary school	F	2 doses (0,6 m)	Gardasil (4v)	School-based				
				2020	Expanded to boys and catch up	Grade 6 F and 11-14 M	F and M	2 doses (0,6 m)	Gardasil (4v)	School-based				
				2021	Routine	12	F and M	2 doses (0,6 m)	Gardasil (4v)	School-based				
	Bolivia (Plurination	2017	2024	current		10	F and M	1 dose	Gardasil (4v)	School-based/health facilities (unschooled)	free	EF	WHO/official web	1.https://immunizationdata.who.int/global/wiise-detail-page/human-papillomavirus-(hpv)-vaccination-coverage?CODE=BOL&ANTIGEN=
				2017	Introduction	10-12	F	2 doses (0,6 m)	Gardasil (4v)					

	al State of)				and catch-up									&YEAR=<br>2.https://www.minsalud.gob.bo/2277-mas-de-siete-mil-brigadas-medicas-se-desplazaran-a-las-unidades-educativas-para-la-vacunacion-contra-el-vph?<br>3.https://www.gov.br/saude/pt-br/centrais-de-conteudo/publicacoes/notas-tecnicas/2024/nota-tecnica-no-41-2024-cgici-dpni-svsa-ms
				2018	Routine	10	F	2 doses (0,6 m)	Gardasil (4v)					
				2023	Dose reduction	10	F	1 dose	Gardasil (4v)					
				2024	Expanded to boys	10	F and M	1 dose	Gardasil (4v)					
	Cabo Verde	2021	2023	current		10	F and M	1 dose	Cervarix (2v)	School-based	NA	EF	WHO/official web	1.https://hpvcentre.net/statistics/reports/CPV.pdf<br>2.https://unsdg.un.org/latest/stories/girls-today-women-tomorrow-hpv-vaccine-sets-new-course-women-and-girls-cabo-verde<br>3.https://cdn.who.int/media/docs/default-source/country-profiles/cervical-cancer/cervical-cancer-cpv-2021-country-profile-en.pdf?sfvrsn=b14fba97_36&download=true<br>4.https://immunizationdata.who.int/global/wiise-detail-page/vaccination-schedule-for-human-papilloma-virus?ISO_3_CODE=CPV&TARGETPOP_GENERAL=
				2021	Introduction	10	F	2 doses	Cervarix (2v)					
				2023	Dose reduction and expanded to boys	10	F and M	1 dose	Cervarix (2v)					
	Cambodia	2023	NA			9	F	1 dose	Cervarix (2v)	School-based	free	GF and EF	WHO/official web/UNICEF	1.https://www.who.int/cambodia/news/detail/22-01-2017-cambodia-introduces-hpv-vaccines<br>2.https://www.nis.gov.kh/nis/Policy_Brief/G2_D2P-Policy%20Brief_Cervical%20cancer%20Eng-final%20Signed.pdf;<br>3.https://www.unicef.org/cambodia/press-releases/life-saving-hpv-vaccine-introduced-nationwide-routine-immunization-schedule-prevent
	Cameroon	2020	2023	current		9	F and M	1 dose	Gardasil (4v)	Mixed (schools and health facilities)	free	EF	WHO/official web/pubmed	1.https://immunizationdata.who.int/global/wiise-detail-page/vaccination-schedule-for-human-papilloma-virus?ISO_3_CODE=CMR&TARGETPOP_GENERAL=<br>2.https://www.minsante.cm/site/?q=en/node/750<br>3.https://www.frontiersin.org/journals/public-health/articles/10.3389/f
				2020	Introduction	9	F	2 doses (0,6 m)	Gardasil (4v)					
				2023	Dose reduction and expanded to	9	F and M	1 dose	Gardasil (4v)					

					boys									pubh.2021.748910/full<br>4.https://gh.bmj.com/content/8/Suppl_10/A104.1
	Côte d'Ivoire	2019	2023	current		9	F	1 dose	Gardasil (4v)	School-based	free	GF and EF	WHO/Gavi/UNICF/IVAC/pubmed	1.https://immunizationdata.who.int/global/wiise-detail-page/vaccination-schedule-for-human-papilloma-virus?ISO_3_CODE=CIV&TARGETPOP_GENERAL=<br>2.https://www.gavi.org/news/media-room/gavi-and-cote-divoire-commit-successful-programmatic-and-financial-transition<br>3.https://www.unicef.org/cotedivoire/recits/le-vaccin-qui-sauve-la-vie-des-filles%E2%80%AF<br>4.https://hpvcentre.net/statistics/reports/CIV.pdf<br>5.https://www.ncbi.nlm.nih.gov/pmc/articles/PMC7957340/
				2019	Introduction and catch-up	9-14	F	2 doses	Gardasil (4v)					
				2023	Dose reduction	9	F	1 dose	Gardasil (4v)					
	Eswatini	2023	NA			9-14	F	2 doses (0,6 m)	Gardasil (4v)	School-based	free	GF	WHO/Gavi	1.https://immunizationdata.who.int/global/wiise-detail-page/vaccination-schedule-for-human-papilloma-virus?ISO_3_CODE=SWZ&TARGETPOP_GENERAL=<br>2.https://www.afro.who.int/countries/eswatini/news/eswatini-receives-usd-16m-grant-gavi-support-hpv-vaccine-introduction<br>3.https://www.afro.who.int/countries/eswatini/news/launching-hpv-vaccination<br>4.https://www.gavi.org/vaccineswork/hpv-vaccine-rolls-out-eswatini
	Honduras	2016	2024	current		11-15	F	1 dose	Gardasil (4v)	School-based	free	EF	WHO/UNICF/IVAC	1.https://immunizationdata.who.int/global/wiise-detail-page/vaccination-schedule-for-human-papilloma-virus?ISO_3_CODE=HND&TARGETPOP_GENERAL=<br>2.https://www.unicef.org/honduras/historias/key-healthier-tomorrows<br>3.https://www.google.com/url?sa=t&rct=j&q=&esrc=s&source=web&
				2016	Introduction	11-15	F	2 doses (0,6 m)	Gardasil (4v)					

				2024	Dose reduction	11-15	F	1 dose	Gardasil (4v)					cd=&ved=2ahUKEwirlu6W8ZmHAxUzsVYBHU1xCkkQFnoECBEQAQ&url=https%3A%2F%2Fwww.unicef.org%2Fhonduras%2Fhistorias%2Fkey-healthier-tomorrows&usg=AOvVaw23pJPPi1Mwcl8xQZbHiWFZ&opi=89978449<br>4.https://hpvcentre.net/statistics/reports/HND_FS.pdf
	Kenya	2019	NA			10-14	F	2 doses (0,6 m)	Gardasil (4v)	Health facility-based	free	GF and EF	official web/Gavi	1.http://guidelines.health.go.ke:8000/media/Kenya_National_Immunization_Policy_Guidelines_Version_signed.pdf<br>2.https://www.globalcitizen.org/en/content/women-championing-hpv-vaccine-kenya/?gad_source=1&gclid=EAIaIQobChMIr5H_4_WZhwMV72APAh2NuQyFEAAYASAAEgIgD_D_BwE<br>3.https://www.gavi.org/news/media-room/kenya-introduces-cervical-cancer-vaccine-nationally?gad_source=1&gclid=EAIaIQobChMIr5H_4_WZhwMV72APAh2NuQyFEAAYAiAAEgIpBfD_BwE
	Kiribati	2023	NA			9	F	1 dose	Gardasil (4v)	NA	NA	NA	WHO/official web/IVAC	1.https://immunizationdata.who.int/global/wiise-detail-page/vaccination-schedule-for-human-papilloma-virus?ISO_3_CODE=KIR&TARGETPOP_GENERAL=<br>2.https://www.google.com/url?sa=t&rct=j&q=&esrc=s&source=web&cd=&cad=rja&uact=8&ved=2ahUKEwil_ZPw9ZmHAxU4r1YBHYXFDV4QFnoECCoQAQ&url=https%3A%2F%2Fuprdoc.ohchr.org%2Fuprweb%2Fdownloadfile.aspx%3Ffilename%3D7466%26file%3DEnglishTranslation&usg=AOvVaw0af4jdh39AMJeC0exWEWnQ&opi=89978449<br>3.https://hpvcentre.net/statistics/reports/KIR.pdf

	Kyrgyzstan	2022	NA			9-14	F	2 doses (0,6 m)	Gardasil (4v)	School-based	NA	EF	WHO	1.https://www.who.int/azerbaijan/news/item/15-02-2023-kyrgyzstan-joins-european-cervical-cancer-prevention-week<br>2.https://www.who.int/about/accountability/results/who-results-report-2020-mtr/country-story/2023/kyrgyzstan-s-successful-drive-to-introduce-hpv-vaccine-cuts-cervical-cancer-risk<br>3.https://immunizationdata.who.int/global/wiise-detail-page/vaccination-schedule-for-human-papilloma-virus?ISO_3_CODE=KGZ&TARGETPOP_GENERAL=
	Lao People's Democratic Republic	2020	NA			10-14	F	2 doses (0,12 m)	Gardasil (4v)	School-based	free	GF and EF	WHO/Gavi	1.https://www.who.int/laos/news/detail/04-03-2020-cervical-cancer-vaccine-introduced-in-lao-pdr<br>2.https://immunizationdata.who.int/global/wiise-detail-page/vaccination-schedule-for-human-papilloma-virus?ISO_3_CODE=LAO&TARGETPOP_GENERAL=<br>3.https://www.gavi.org/vaccineswork/fighting-cervical-cancer-lao-pdr
	Lesotho	2022	NA			9-14	F	2 doses (0,6 m)	NA	School-based	free	EF	Cavi/official news web	1.https://www.gavi.org/vaccineswork/lesotho-mothers-welcome-reintroduction-hpv-vaccine<br>2.https://www.gavi.org/programmes-impact/country-hub/africa/lesotho<br>3.https://www.africa-newsroom.com/press/lesotho-health-minister-urges-for-human-papillomavirus-hpv-vaccination?lang=en<br>4.https://allafrica.com/stories/202402110143.html
	Mauritania	2021	NA			9	F	2 doses (0,6 m)	Gardasil (4v)	NA	NA	NA	WHO/IVAC	1.https://immunizationdata.who.int/global/wiise-detail-page/vaccination-schedule-for-human-papilloma-virus?ISO_3_CODE=MRT&TARGETPOP_GENERAL=<br>1.https://cdn.who.int/media/docs/default-source/country-profiles/cervical-cancer/cervical-cancer-mrt-2021-country-profile-en.pdf?sfvrsn=73

														69a73_13<br>2.https://hpvcentre.net/statistics/reports/MRT.pdf
	Micronesia (Federated States of)	2010	NA			10-11	F	2 doses (0,6 m)	Gardasil 9 (9v)	School-based	NA	NA	WHO/official web	1.https://immunizationdata.who.int/global/wiise-detail-page/vaccination-schedule-for-human-papilloma-virus?ISO_3_CODE=SLB&TARGETPOP_GENERAL=<br>2.https://ftp.cdc.gov/pub/Publications/Cancer/ccc/fsm_ccc_plan-508.pdf
	Mongolia	2024	NA			11	F and M	1 dose	Gardasil (4v)	School-based	free	GF and EF	WHO/Gavi/official news web	1.https://www.who.int/mongolia/news/detail/22-04-2025-mongolia-launches-national-programme-to-eliminate-cervical-cancer;<br>2.https://immunizationdata.who.int/global/wiise-detail-page/vaccination-schedule-for-human-papilloma-virus?ISO_3_CODE=&TARGETPOP_GENERAL=<br>3.https://www.gavi.org/vaccineswork/mongolia-facing-high-rates-cervical-cancer-launch-massive-human-papillomavirus;<br>4.https://english.news.cn/20250124/d2e1a12beaeb4cc1b5e7e66068caa1eb/c.html;
	Morocco	2022	NA			11	F	2 doses (0,6 m)	Gardasil (4v)	School-based	free	GF and EF	official web/IVAC	1.https://sehati.gov.ma/uploads/CALENDRIER_VACCINATION_2023.pdf<br>2.https://hpvcentre.net/statistics/reports/MAR_FS.pdf

	Myanmar	2020	NA			9-10	F	2 doses (0,12 m)	Gardasil (4v)	School-based	free	GF and EF	WHO/official web/Gavi	1.https://immunizationdata.who.int/global/wiise-detail-page/vaccination-schedule-for-human-papilloma-virus?ISO_3_CODE=MMR&TARGETPOP_GENERAL= 2.https://www.mohs.gov.mm/ckfinder/connector?command=Proxy&lang=en&type=Main&currentFolder=%2FPublications%2FDPH%2FEPI%2F&hash=a6a1c319429b7abc0a8e21dc137ab33930842cf5&fileName=CervixCancer_HPV%20(20-10-2020).pdf 3.https://www.gavi.org/news/media-room/myanmar-introduces-cervical-cancer-vaccine-nationally-despite-covid-19-challenges 4.https://www.gavi.org/vaccineswork/myanmar-celebrates-launch-hpv-vaccine 5.https://www.moi.gov.mm/moi:eng/news/1745
	Nicaragua	2023	NA			10-14	F	2 doses (0,6 m)	Cecolin (2v)	Mixed (schools and health fairs, door-to-door visits, Health facilities)	NA	NA	WHO/official web/PATH	1.https://immunizationdata.who.int/global/wiise-detail-page/vaccination-schedule-for-human-papilloma-virus?ISO_3_CODE=NIC&TARGETPOP_GENERAL= 2.https://www.minsa.gob.ni/index.php/campanas/plan-escuelas-saludables-2024 3.https://www.paho.org/es/noticias/6-11-2023-nicaragua-inicia-vacunacion-contra-virus-papiloma-humano-para-garantizar-futuro
	Nigeria	2023	NA			9-14	F	1 dose	Gardasil (4v)	Mixed (schools and community centres)	free	GF and EF	WHO/official web/UNICF	1.https://immunizationdata.who.int/global/wiise-detail-page/vaccination-schedule-for-human-papilloma-virus?ISO_3_CODE=NGA&TARGETPOP_GENERAL= 2.https://publichealth.jhu.edu/ivac/2024/one-dose-at-a-time-mobilizing-to-eliminate-cervical-cancer-in-nigeria 3.https://www.unicef.org/nigeria/press-releases/nigeria-vaccinate-77-million-girls-against-leading-cause-cervical-cancer

	Philippines	2016	NA			9 (Grade 4 in primary school)	F	2 doses (0,6 m)	Gardasil (4v)	School-based	free	GF	WHO/official web	1.https://immunizationdata.who.int/global/wiise-detail-page/vaccination-schedule-for-human-papilloma-virus?ISO_3_CODE=PHL&TARGETPOP_GENERAL= 2.https://www.pidsphil.org/home/wp-content/uploads/2024/03/CHILDHOOD-IMMUNIZATION-SCHEDULE-2024.pdf 3.https://ncroffice.doh.gov.ph/LatestNews/Details/34 4.https://www.pids.gov.ph/details/news/in-the-news/accelerate-hpv-vaccination-to-save-more-lives-solon 5.https://pmc.ncbi.nlm.nih.gov/articles/PMC9251542/?utm_source=chatgpt.com 5.https://www.pidsphil.org/home/themencode-pdf-viewer/?file=https://www.pidsphil.org/home/wp-content/uploads/2024/11/2025-PIDSP-Immunization-Calendar.pdf#zoom=auto 6.https://www.pna.gov.ph/articles/1195526?utm_source=chatgpt.com
	Samoa	2022	2023	Current		13 (Grade 8 in primary school)	F	1 dose	Cervarix (2v)	School-based	free	GF and EF	official web	1.https://www.health.gov.ws/wp-content/uploads/2023/02/National-Immunization-Policy-2020-2025.pdf 2.https://www.health.gov.ws/wp-content/uploads/2024/06/MASTER-COPY-CERVICAL-CANCER-ELIMINATION-STRATEGY.pdf
				2022		8-13	F	1 dose	Cervarix (2v)					
				2023		13 (Grade 8 in primary school)	F	1 dose	Cervarix (2v)					
	Sao Tome and Principe	2021	2023	Current		NA	F	1 dose	NA	NA	NA	NA	WHO/Gavi	1.https://immunizationdata.who.int/global/wiise-detail-page/human-papillomavirus-(hpv)-vaccination-coverage?CODE=STP&ANTIGEN=&YEAR= 2.https://www.gavi.org/vaccineswork/preventing-cervical-cancer-mozambique-sao-tome-and-principe-and-guinea-bissau;
				2021	Introduction	NA	F	2 doses	NA					
				2023	Dose reduction	NA	F	1 dose	NA					

	Senegal	2018	NA			9	F	2 doses (0,6~12 m）	Gardasil (4v)	School-based/Health facilities	free	GF and EF	WHO/UICC/IVAC/pubmed	1.https://immunizationdata.who.int/global/wiise-detail-page/vaccination-schedule-for-human-papilloma-virus?ISO_3_CODE=SEN&TARGETPOP_GENERAL=<br>2.https://www.technet-21.org/en/resources/case-study/senegal-plan-dintroduction-du-vaccin-contre-le-virus-du-papillome-humain<br>2.https://www.uicc.org/case-studies/promoting-integrated-approaches-cervical-cancer-senegal<br>3.https://hpvcentre.net/statistics/reports/SEN.pdf<br>4.https://hpvcentre.net/statistics/reports/SEN_FS.pdf<br>5.https://www.sciencedirect.com/science/article/pii/S0264410X21010756<br>6.https://journals.plos.org/globalpublichealth/article?id=10.1371/journal.pgph.0000130
	Solomon Islands	2019	NA			9-14	F	2 doses	Gardasil 9 (9v)	School-based/community outreach strategy (unschooled)	NA	EF	WHO/gavi	1.https://immunizationdata.who.int/global/wiise-detail-page/vaccination-schedule-for-human-papilloma-virus?ISO_3_CODE=SLB&TARGETPOP_GENERAL=<br>2.https://www.who.int/westernpacific/about/how-we-work/pacific-support/news/detail/19-09-2022-solomon-islands-boosts-efforts-to-prevent-and-treat-cervical-cancer<br>3.https://www.who.int/westernpacific/news-room/feature-stories/item/protecting-women-in-solomon-islands-from-cervical-cancer<br>4.https://www.who.int/newzealand/news/detail/17-07-2025-hpv-vaccine-rollout-begins--a-life-saving-shot-for-the-girls-of-solomon-islands?utm_source=chatgpt.com<br>5.https://ncirs.org.au/solomon-islands-big-catch-hpv-vaccination-campaign-kicks?utm_source=chatgpt.com

														6.https://www.gavi.org/sites/default/files/document/2021/cMYP%20Solomon%20Islands%202016-2020.pdf<br>7.https://www.mdpi.com/2076-393X/9/11/1355?utm_source=chatgpt.com
	Sri Lanka	2017	NA			10-11	F	2 doses (0,6 m)	Gardasil (4v)	School-based/Health facilities (unschooled)	free	GF and EF	official web/pubmed	1.https://nccp.health.gov.lk/storage/post/pdfs/Final%20guideline.pdf<br>2.https://www.ncbi.nlm.nih.gov/pmc/articles/PMC10877402/
	Timor-leste	2024	2024			11-14	F	1 dose	Gardasil (4v)	School-based	free	GF and EF	WHO/official web/Gavi	1.https://immunizationdata.who.int/global/wiise-detail-page/vaccination-schedule-for-human-papilloma-virus?ISO_3_CODE=&TARGETPOP_GENERAL=<br>2.https://ncirs.org.au/timor-leste-rolls-out-hpv-vaccination-program;<br>3.https://timor-leste.gov.tl/?p=38589&lang=en&n=1;<br>4.https://www.gavi.org/news/media-room/61374-girls-timor-leste-receive-hpv-vaccine-combat-cervical-cancer;4.https://ncirs.org.au/timor-leste-rolls-out-hpv-vaccination-program;5.https://www.who.int/timorleste/news/detail/16-10-2024-timor-leste-s-rapid-hpv-vaccine-drive--over-90--girls-immunized-in-just-3-weeks
	United Republic of	2018	2024	Current		9	F	1 dose	Gardasil (4v)	Mixed (schools, health facilities and	free	GF and EF	WHO/official web/Gavi/pubme	https://www.moh.go.tz/search?q=HPV+dhidi<br>2.https://www.afro.who.int/countries/united-republic-of-tanzania/news
				2018	Introduction	14	F	2 doses (0,6 m)	Gardasil (4v)					

	Tanzania			2024.4.22-4.26	Catch-up	9-14	F	1 dose	Gardasil (4v)	community centres)			d	/over-5-million-girls-tanzania-receive-hpv-vaccine-combat-cervical-cancer<br>3.https://www.ncbi.nlm.nih.gov/pmc/articles/PMC9601816/
				2024.4.27	Routine	9	F	1 dose	Gardasil (4v)					
	Uzbekistan	2019	NA			9	F	2 doses (0,6 m)	Gardasil (4v)	School-based	free	GF and EF	UNICF	1.https://www.unicef.org/uzbekistan/en/stories/cervical-cancer-uzbekistan
	Vanuatu	2023 （**partial**）	NA			9-13	F	1 dose	NA	Mixed (schools, health facilities and community centres)	free	GF and EF	WHO/official web/IVAC/UNICF	1.https://immunizationdata.who.int/global/wiise-detail-page/vaccination-schedule-for-human-papilloma-virus?ISO_3_CODE=VUT&TARGETPOP_GENERAL=FEMALE<br>2.https://www.kirby.unsw.edu.au/news/vanuatu-becomes-first-pacific-set-path-towards-cervical-cancer-elimination<br>3.https://hpvcentre.net/statistics/reports/VUT.pdf<br>4.https://www.unicef.org/pacificislands/press-releases/pacific-children-get-health-boost-government-immunization-commitments<br>5.https://www.cancercouncil.com.au/news/eliminate-cervical-cancer-png-vanuatu/
	Zambia	2019	2023	Current	Routine	9	F	1 dose	Gardasil (4v)	Mixed (schools, health facilities and community centres)	free	GF and EF	WHO/Gavi/UNICF/pubmed	1.https://www.technet-21.org/en/resources/guidance/hpv-guidelines-for-vaccinators<br>2.https://www.gavi.org/vaccineswork/zambia-launches-major-hpv-vaccine-campaign?gad_source=1&gclid=EAIaIQobChMI7Mfe5PfBhwMVKlsPAh0xjw25EAAYASAAEgLN_vD_BwE<br>3.https://www.unicef.org/zambia/press-releases/ministry-health-launches-hpv-multi-age-cohort-mac-vaccination-campaign<br>4.https://www.ncbi.nlm.nih.gov/pmc/articles/PMC10312939/
					Risk-group	9 (with HIV)	F	3 doses (0,6,12 m)	Gardasil (4v)					
				2019	Introduction	14	F	2 doses (0,6 m)	Gardasil (4v)					
				2023	Catch-up	9-14	F	2 doses (0,6 m)	Gardasil (4v)					
				2024	Routine	9	F	1 dose	Gardasil (4v)					
					Risk-group	9 (with HIV)	F	3 doses (0,6,12 m)	Gardasil (4v)					

	Zimbabwe	2018	2019	current		10 (Grade 5 in primary school)	F	2 doses (0,12 m)	Cervarix (2v)	School-based/health facilities (unschooled)	free	GF and EF	WHO/official web/gavi/pubmed	1.https://immunizationdata.who.int/global/wiise-detail-page/vaccination-schedule-for-human-papilloma-virus?ISO_3_CODE=ZWE&TARGETPOP_GENERAL= 2.https://www.technet-21.org/en/resources/guidance/hpv-field-guide 3.https://www.gavi.org/news/media-room/cervical-cancer-vaccine-introduced-zimbabwe?gad_source=1&gclid=EAIaIQobChMI48jg4vzBhwMVUGwPAh023Q5AEAAYASAAEgKasvD_BwE 4.https://www.jsi.com/resource/hpv-vaccination-coverage-in-three-districts-in-zimbabwe-following-national-introduction-of-012-month-schedule-among-10-to-14-year-old-girls/ 5.https://karger.com/int/article/67/1/55/898532/Human-Papillomavirus-Genotype-Distribution 5.https://journals.plos.org/globalpublichealth/article?id=10.1371/journal.pgph.0000101
				2018	Introduction and catch-up	10-14	F	2 doses (0,12 m)	Cervarix (2v)					
				2019	Routine	10 (Grade 5 in primary school)	F	2 doses (0,12 m)	Cervarix (2v)					
LICs	Burkina Faso	2022	NA			9	F	1 dose	Gardasil (4v)	Mixed (schools and health facilities)	free	GF and EF	WHO	1.https://www.who.int/azerbaijan/news/item/15-02-2023-kyrgyzstan-joins-european-cervical-cancer-prevention-week
	Eritrea	2022	NA			9-14	F	2 doses (0,6 m)	Gardasil (4v)	Mixed (schools and Health facilities for remote villages)	free	EF	WHO/official web/Gavi	1.https://immunizationdata.who.int/global/wiise-detail-page/vaccination-schedule-for-human-papilloma-virus?ISO_3_CODE=ERI&TARGETPOP_GENERAL= 2.https://immunizationdata.who.int/global/wiise-detail-page/human-papillomavirus-(hpv)-vaccination-coverage?CODE=ERI&ANTIGEN=&YEAR= 3.https://shabait.com/2023/07/01/hpv-vaccination-campaign-in-eritrea/ 4.https://www.gavi.org/programmes-impact/country-hub/africa/eritrea 5.https://www.gavi.org/vaccineswork/eritrea-touts-screening-and-hpv-vaccines-drive-down-cervical-cancer-rates

	Ethiopia	2018	2022	current		14	F	1 dose	Gardasil (4v)	Mixed (schools and Health facilities for vulnerable communities)	free	EF	WHO/official web	1.https://www.afro.who.int/countries/ethiopia/news/human-papillomavirus-vaccination-ethiopia-protects-over-63-million-girls-cervical-cancer<br>2.https://www.afro.who.int/countries/ethiopia/news/ethiopia-vaccinate-over-18-million-girls-against-human-papillomavirus-part-drive-prevent-cervical<br>3.https://www.moh.gov.et/en/mnch-epi<br>4.https://www.moh.gov.et/en/initiatives-4-col/Expanded_Program_on_Immunization
				2018	Introduction	14	F	2 doses (0,6 m)	Gardasil (4v)					
				2024	Dose reduction	14	F	1 dose	Gardasil (4v)					
	Gambia	2019	2023	current		9-14	F	1 dose	Gardasil (4v)	Mixed (schools and community centres)	NA	EF	WHO/official web/web of science	1.https://immunizationdata.who.int/global/wiise-detail-page/vaccination-schedule-for-human-papilloma-virus?ISO_3_CODE=GMB&TARGETPOP_GENERAL=<br>2.https://immunizationdata.who.int/global/wiise-detail-page/introduction-of-hpv-(human-papilloma-virus)-vaccine?ISO_3_CODE=GMB&YEAR=<br>3.https://immunizationdata.who.int/global/wiise-detail-page/human-papillomavirus-(hpv)-vaccination-coverage?CODE=GMB&ANTIGEN=&YEAR=<br>4.https://www.iccp-portal.org/system/files/plans/GMB_B5_CSP%20of%20The%20Gambia-%20%202016%20%E2%80%93%202020%20Final.pdf<br>5.https://www.sciencedirect.com/science/article/abs/pii/S0264410X2300508X?via%3Dihub
				2019	Introduction	9-14	F	2 doses (0,12 m)	Gardasil (4v)					
				2023	Dose reduction	9-14	F	1 dose	Gardasil (4v)					

	Liberia	2019	NA			9-14	F	2 doses (0,6 m)	Gardasil (4v)	Mixed (schools and health facilities)	NA	GF and EF	WHO/official news web	1.https://www.afro.who.int/countries/liberia/news/liberia-rewriting-cervical-cancer-narrative-one-dose-time<br>2.https://www.afro.who.int/news/liberia-successfully-introduces-vaccine-against-cervical-cancer<br>3.https://www.who.int/about/accountability/results/who-results-report-2020-mtr/country-story/2021/liberia<br>4.https://immunizationdata.who.int/global/wiise-detail-page/vaccination-schedule-for-human-papilloma-virus?ISO_3_CODE=LBR&TARGETPOP_GENERAL=<br>5.https://allafrica.com/stories/202010230190.html
	Malawi	2019	NA			9	F	2 doses (0,6 m)	Gardasil (4v)	Mixed (schools, health facilities and community centres)	free	GF and EF	official web/pubmed	1.https://www.nyasatimes.com/malawi-launches-hpv-vaccine-campaign-minister-muluzi-says-cervical-cancer-leading-cause-of-womens-deaths/<br>2.https://www.sciencedirect.com/science/article/pii/S2590136223000566#b0045
	Mali	2024	2024			10	F	1 dose	Gardasil (4v)	Mixed (fixed, advanced, mobile facilities for vulnerable communities)	free	GF and EF	WHO/official web	1.https://immunizationdata.who.int/global/wiise-detail-page/vaccination-schedule-for-human-papilloma-virus?ISO_3_CODE=&TARGETPOP_GENERAL=<br>2.http://www.sante.gov.ml/index.php/actualites/item/9429-lutte-contre-le-cancer-de-col-de-l-uterus-le-vaccin-desormais-disponible-au-mali-pour-les-filles-de-10-ans;<br>3.https://www.vax-before-travel.com/2024/11/26/another-sahelian-country-offers-single-dose-hpv-vaccination-0;

	Mozambique	2021	2022	current		9-14	F	2 doses (0,6 m)	Cervarix (2v)	School-based/other strategy (door-to-door approach, mobile outreach, and vaccination at fixed facilities)	free	GF and EF	WHO/official web/pubmed	1.https://immunizationdata.who.int/global/wiise-detail-page/vaccination-schedule-for-human-papilloma-virus?ISO_3_CODE=MOZ&TARGETPOP_GENERAL= 2.https://www.isglobal.org/en/healthisglobal/-/custom-blog-portlet/mozambique-y-la-vacuna-contra-el-papiloma-humano-de-recomendacion-a-realidad-en-dos-decadas 3.https://www.afro.who.int/countries/mozambique/news/mozambique-boosts-hpv-vaccination-reach-high-coverage?utm_source=chatgpt.com 4.https://www.isglobal.org/documents/10179/25254/Mozambique-Human+Papillomavirus+Infection+and+Cervical+Cancer.pdf/d3cb8884-a8fc-4989-b143-88242b593cda 5.https://bmcpublichealth.biomedcentral.com/articles/10.1186/s12889-019-7793-y
				2021	Introduction	9-14	F	2 doses (0,6 m)	Cervarix (2v)					
				2022	Catch-up	born in 2012	F	2 doses (0,6 m)	Cervarix (2v)					
	Rwanda	2011	2015	current		12	F	2 doses (0,6 m)	Gardasil (4v)	School-based	free	GF and EF	official web/pubmed	1.https://rbc.gov.rw/fileadmin/user_upload/annoucement/Vaccine%20calendar.pdf 2.https://rbc.gov.rw/cervicalcancer/spip.php?article9 3.https://www.ncbi.nlm.nih.gov/pmc/articles/PMC7221340/
				2011	Introduction	Grade 6 in primary school	F	3 doses (0,2,6 m)	Gardasil (4v)	School-based/Health facilities (unschooled)	free	EF		
				2012	catch-up	Grade 3 in secondary school	F	3 doses (0,2,6 m)	Gardasil (4v)					
				2014	catch-up	12-14	F	3 doses (0,2,6 m)	Gardasil (4v)					
				2015	Dose reduction	12	F	2 doses (0,6 m)	Gardasil (4v)			GF and EF		
	Sierra Leone	2022	NA			10	F	2 doses	Cervarix (2v)	School-based	free	GF	official web	https://dhse.gov.sl/sierra-leone-introduces-hpv-vaccine-to-protect-girls-from-cervical-cancer/

	Togo	2023	NA			9-14	F	1 dose	Cervarix (2v)	School-based	free	EF and SHI	WHO/Gavi/pubmed/official news web	1.https://immunizationdata.who.int/global/wiise-detail-page/human-papillomavirus-(hpv)-vaccination-coverage?CODE=TGO&ANTIGEN=&YEAR=<br>2.https://www.gavi.org/news/media-room/togo-introduces-human-papillomavirus-vaccine-protect-adolescent-girls-leading<br>3.https://www.sciencedirect.com/science/article/pii/S0264410X21014663<br>4.https://www.afro.who.int/news/africa-immunization-advisory-group-urges-single-dose-hpv-vaccine-adoption-advance-vaccination?utm_source=chatgpt.com
	Uganda	2015	NA			10	F	2 doses (0,6 m)	Gardasil (4v)	School-based	free	GF and EF	official web/UNICF	1.https://www.health.go.ug/wp-content/uploads/2019/11/UNEPI-Immunization-guidelines.pdf<br>2.https://www.unicef.org/uganda/media/4516/file/HPV%20fact%20sheet%20-%20A4.pdf

Notes:Upper-MICs:upper-middle-income countries; Lower-MICs:lower-middle-income countries;LICs:low-income countries; ICP:Immunocompromised person; F:female; M:male; FBiH-CS:Federation of Bosnia and Herzegovina,Canton Sarajevo; FBiH-other:Federation of Bosnia and Herzegovina, Elsewhere; RS:Federation of Bosnia and Herzegovina,Republika Srpska; m:months; ys: years; GF:Goverment fund; EF:External fund;SHI:Social health insurance;WHO:World Health Organization;Gavi:The Global Alliance for Vaccines and Immunisation;UNICEF: United Nations Children's Fund;UNFPA: United Nations Population Fund;UICC: Union for International Cancer Control;IVAC: International Vaccine Access Center.

Supplementary Table 4. Timeline of HPV Vaccine Strategy Adoption into National Immunization Programs in LMICs

Income level	2008	2009	2010	2011	2012	2013	2014	2015	2016	2017	2018	2019	2020	2021	2022	2023	2024
Upper-MICs	Mexico（partial）	Marshall Islands<br>North Macedonia<br>Malaysia（partial）	Malaysia	Argentina	Colombia<br>Mexico	Fiji<br>Libya<br>Paraguay<br>Suriname<br>Brazil（partial）	Brazil<br>Ecuador<br>South Africa	Botswana<br>Peru	Belize<br>Turkmenistan<br>Indonesia（partial）	Armenia<br>Dominican Republic<br>Jamaica<br>Mauritius<br>Saint Vincent and the Grenadines<br>Thailand<br>Republic of Moldova（partial）	Guatemala	Dominica<br>Georgia<br>Grenada<br>Maldives<br>Saint Lucia	El Salvador	Cabo Verde<br>Tuvalu	Albania<br>Montenegro<br>Samoa<br>Serbia<br>Tonga	Bosnia and Herzegovina<br>Indonesia	Kazakhstan<br>Mongolia
Lower-MICs		Bhutan	Micronesia (Federated States of)						Honduras<br>Philippines（partial）	Bolivia (Plurinational State of)<br>Sri Lanka	Senegal<br>United Republic of Tanzania<br>Zimbabwe<br>Ethiopia	Côte d'Ivoire<br>Kenya<br>Solomon Islands<br>Uzbekistan<br>Zambia	Cameroon<br>Lao People's Democratic Republic<br>Myanmar	Mauritania<br>Sao Tome and Principe	Kyrgyzstan<br>Lesotho<br>Morocco	Cambodia<br>Eswatini<br>Kiribati<br>Nicaragua<br>Bangladesh（partial）<br>Nigeria（partial）<br>Vanuatu（partial）	Bangladesh<br>Timor-Leste<br>Vanuatu
LICs				Rwanda				Uganda				Gambia<br>Liberia<br>Malawi		Mozambique	Burkina Faso<br>Eritrea<br>Sierra Leone	Togo	Mali

Notes: Upper-MICs:upper-middle-income countries; Lower-MICs:lower-middle-income countries;LICs:low-income countries.

Supplementary Table 5. Summary characteristics of cooperation mechanism

Income level	Country（region）	Government Interdepartmental	Government-International-NGO	Public-Private Partnership	Community Engagement
Upper-MICs	Albania	-	UNFPA	-	-
	Argentina	MOH-MOE	-	-	-
	Armenia	-	-	-	-
	Belize	MOH-MOE	-	-	-
	Bosnia and Herzegovina	-	-	-	-
	Botswana	MOH-MOE	-	vaccine manufacturer	-
	Brazil	MOH-MOE	PAHO	Merck	-
	Bulgaria	MOH-MOE	-	-	-
	Colombia	MOH-MOE	-	-	-
	Costa Rica	MOH-MOE	-	-	-
	Dominica	MOH-MOE	-	-	Healthcare providers and Parentsd
	Dominican Republic	MOH-MOE	PAHO	-	-
	Ecuador	MOH-MOE	-	-	-
	El Salvador	MOH-MOE	World Bank	-	-
	Fiji	MOH-MOE	Australia Government	-	-
	Georgia	-	Gavi (former support), WHO, UNFPA	-	-
	Grenada	MOH-MOE-MOSD	-	-	-
	Guatemala	MOH-MOE	-	-	-
	Indonesia	MOH-MOE	Gavi (Middle-Income Countries Approach)	-	-
	Jamaica	MOH-MOE	-	-	-
	Kazakhstan	-	-	-	-
	Libya	-	UNICIF,WHO	-	-
	Malaysia	MOH-MOE	-	-	-
	Maldives	MOH-MOE	-	-	-
	Marshall Islands	MOH-MOE	-	-	-
	Mauritius	MOH-MOE	-	-	-
	Mexico	MOH-MOE	-	-	-
	Montenegro	-	WHO	-	-
	North Macedonia	MOH-MOE	-	-	-
	Palau	-	-	-	-
	Paraguay	MOH-MOE	-	-	-
	Peru	MOH-MOE	PAHO	Merck	-
	Republic of Moldova	-	UNICIF,UNFPA,WHO	-	-
	Saint Lucia	MOH-MOE	-	-	-
	Saint Vincent and the Grenadines	MOH-MOE	WHO	-	-
	Serbia	-	UNICIF,WHO	-	-
	South Africa	MOH-MOE	-	-	-

	Suriname	MOH-MOE	-	-	-
	Thailand	MOH-MOE	-	-	-
	Tonga	MOH-MOE	-	-	-
	Turkmenistan	MOH-MOE	WHO	-	-
	Tuvalu	-	-	-	-
Lower-MICs	Bangladesh	MOH-MOE	Gavi (accelerated transition phase), UNICEF	-	-
	Bhutan	MOH-MOE	Australian Cervical Cancer Foundation, UNPFA	-	Community leaders, Parents, Teachers
	Bolivia (Plurinational State of)	MOH-MOE	PAHO	-	-
	Cabo Verde	MOH-MOE	UNICEF	-	-
	Cambodia	MoH-MoEYS-local administrations	Gavi (preparatory transition phase), WHO,UNICEF,and CHAI	-	Teachers and Health volunteers
	Cameroon	MOH-MOE	Gavi (preparatory transition phase)	-	-
	Côte d'Ivoire	MOH-MOE	Gavi (accelerated transition phase)	-	-
	Eswatini	MOH-MOE	-	-	-
	Honduras	MOH-MOE	UNICEF, Canadian Government	-	-
	Kenya	-	Gavi (preparatory transition phase)	-	Teachers and Father
	Kiribati	-	-	-	-
	Kyrgyzstan	MOH-MOE	WHO, Gavi (preparatory transition phase)	-	-
	Lao People's Democratic Republic	MOH-MOE	Gavi (preparatory transition phase)	-	-
	Lesotho	MOH-MOE	Gavi (preparatory transition phase)	-	-
	Mauritania	-	Gavi (preparatory transition phase)	-	-
	Micronesia (Federated States of)	MOH-MOE	-	-	-
	Mongolia	MOH-MOE	UNICEF, WHO	-	-
	Morocco	MOH-MOE	-	-	-
	Myanmar	MOH-MOE	Gavi (preparatory transition phase)	-	-
	Nicaragua	MOH-MOE	-	-	-
	Nigeria	MOH-MOE	Gavi (preparatory transition phase), UNICEF, WHO	-	-
	Philippines	MOH-MOE	-	-	-
	Samoa	MOH-MOE	-	-	-
	Sao Tome and Principe	-	Gavi (accelerated transition phase)	-	-
	Senegal	MOH-MOE	Gavi (preparatory transition phase), UNICEF	-	Community
	Solomon Islands	MOH-MOE	Gavi (preparatory transition phase), UNICEF, WHO, PATH, the Australian Cervical Cancer Foundation and Rotary	-	-

			International		
	Sri Lanka	MOH-MOE	Gavi (preparatory transition phase)	-	-
	Timor-leste	MOH-MOE	Gavi (preparatory transition phase)	-	-
	United Republic of Tanzania	MOH-MOE	Gavi (preparatory transition phase), UNICEF, WHO	-	Community leaders
	Uzbekistan	MOH-MOE	UNICEF	-	-
	Vanuatu	MOH-MOE	Australia Minderoo Foundation	-	Community center
	Zambia	MOH-MOE	Gavi (preparatory transition phase)	-	Community center
	Zimbabwe	MOH-MOE	Gavi (preparatory transition phase), WHO, UNICEF	-	-
LICs	Burkina Faso	MOH-MOE	Gavi (initial self-financing phase), UICC	-	-
	Eritrea	MOH-MOE	Gavi (initial self-financing phase), UNICEF	-	-
	Ethiopia	MOH-MOE	Gavi (initial self-financing phase)	-	-
	Gambia	MOH-MOE	Gavi (initial self-financing phase), UNICEF	-	Community center
	Liberia	MOH-MOE	Gavi (initial self-financing phase), UNICEF,WHO	-	-
	Malawi	MOH-MOE	Gavi (initial self-financing phase)	-	Community center
	Mali	-	Gavi (initial self-financing phase)	-	-
	Mozambique	MOH-MOE	Gavi (initial self-financing phase)	-	Community center
	Rwanda	MOH-MOE	Gavi (initial self-financing phase)	Merck	Women groups, Opinion leaders
	Sierra Leone	MOH-MOE	Gavi (initial self-financing phase)	-	-
	Togo	MOH-MOE	Gavi (initial self-financing phase), UNICEF, WHO, UNFPA	-	-
	Uganda	MOH-MOE	Gavi (initial self-financing phase), WHO,UNICEF,PATH, SIDA	-	-

Notes:Upper-MICs:upper-middle-income countries; Lower-MICs:lower-middle-income countries; LICs:low-income countries; MOH: Ministry of Health; MOE: Ministry of Education; MOSD:Ministry of Social Development; Gavi:the Global Alliance for Vaccines and Immunization, phase source (updated 2026): https://www.gavi.org/partner-countries/programmatic-policies/eligibility-policy;WHO: World Health Organization; UNICEF: the United Nations International Children's Emergency Fund; PATH: the Program for Appropriate Technology in Health; UNFPA:the United Nations Population Fund; Merck:Merck Sharp & Dohme Corporation; CHAI:Clinton Health Access Initiative;UICC: Union for International Cancer Control;Swedish International Development Cooperation Agency.

Supplementary Table 6. Supplementary strategies for reaching underserved adolescents and corresponding 2024 HPV vaccination coverage

Income level	Country（region）	Delivery platform	Detailed strategy	15HPV1_F Coverage (%)	15HPVC_F Coverage (%)	15HPV1_M Coverage (%)	15HPVC_M Coverage (%)
Upper-MICs	Mexico	School based	with Health facilities for unschooled	80	80	-	-
	Belize	School based	with Health facilities for unschooled	67	67	27	27
	Botswana	School based	with Health facilities for unschooled	55	16	-	-
Lower-MICs	Sri Lanka	School based	with Health facilities for unschooled	95	74	-	-
	Zimbabwe	School based	with Health facilities for unschooled	88	39	-	-
	Bolivia (Plurinational State of)	School based	with Health facilities for unschooled	79	69	-	-
	Solomon Islands	School based	with community outreach strategy for unschooled	51	51	-	-
Low-MICs	Rwanda	School based	with Health facilities for unschooled	78	73	-	-
	Mozambique	School based	with other strategy (door-to-door approach, mobile outreach, and vaccination at fixed facilities) for vulnerable communities	-	-	-	-
Lower-MICs	Bangladesh	Mixed	schools and health facilities for unschooled	15	15	-	-
	Nicaragua	Mixed	schools and health fairs, door-to-door visits, Health facilities	-	-	-	-
Low-MICs	Eritrea	Mixed	schools and Health facilities for remote villages	88	99		
	Ethiopia	Mixed	schools and Health facilities for vulnerable communities	60	29	-	-
	Mali	Mixed	fixed, advanced, mobile facilities for vulnerable communities	-	-	-	-

Notes:Upper-MICs:upper-middle-income countries; Lower-MICs:lower-middle-income countries;LICs:low-income countries; -:Not available; 15HPV1_F: HPV Vaccination coverage by age 15,first dose, females; 15HPVC_F: HPV Vaccination coverage by age 15,last dose, females;15HPV1_M: HPV Vaccination coverage by age 15,first dose, males; 15HPVC_M: HPV Vaccination coverage by age 15,last dose, males; HPV vaccination coverage in countries in 2024 was retrieved from WHO website.

Supplementary Table 7. Summary characteristics of implementation barriers

Income level	Country	System barrier	Social-cultural barrier	Knowledge barrier	Resource barrier	Others	Research method	Cite
Upper-MICs	Indonesia	-	Side-effect fears	-	Health care services not accessible in rural areas, High vaccine costs	-	Quant	1
	Mexico	-	Societal attitudes, Fertility concerns	Poor public infomation, Limited health worker knowledge		-	Quali	2
	South Africa	Obtaining informed consent	-	Social media spreading misinformation about HPV vaccination	Vulnerabilities in cold chain capacity	Onsite management of minor adverse events	Mixed	3
Lower-MICs	Bhutan	Vaccine recipient tracking problem	Male vaccine hesitancy	-	Vaccine delivery delay	-	Quali	4
	Cameroon	Weak leadership commitment	Strong opposition from religious leaders			Communication gaps	Quali	5
	Côte d'Ivoire		Vaccine hesitancy	Insufficient communication, poor awareness		-	Quali	5
	Eswatini	-	-	-	-	Communication gap between parents and school authorities	Quali	5
	Kenya	-	-	-	Transport costs, Distance barriers	-	Quali	6
	Nigeria			Low awareness	High cost, lack of convenience and		Mixed	7

					accessibility			
	Senegal	-	-	Rumors, misinformation	-	Insufficient community activities	Quali	[5]
	Tanzania	-	-	-	-	Exclusion of out-of-school girls due to inadequate outreach activities	Quali	[5]
	Uzbekistan	Regulatory barriers	-	-	High cost	-	Quali	[8]
	Zambia	-	-	-	Supply chain issue	Frequent school change of girls	Quali	[5]
	Zimbabwe	-	-	-	Cost and accessibility of vaccines	Transitioning from a multiple age-based to single grade- and age-based target population	Quali	[9]
LICs	Ethiopia	Unstable political situation	-	-	-	Shaky administrative data	Quali	[5]
	Malawi	-	-	Rumors and misconception	-	-	Quali	[5]
	Uganda	-	-	-	Inadequate human and material capacities	-	Quali	[5]

Notes:Upper-MICs:upper-middle-income countries; Lower-MICs:lower-middle-income countries;LICs:low-income countries; -:Not available; Quali:Qualitative research; Quant: Quantitative research.

Supplementary Table 8. Summary characteristics of vaccine hesitancy

Income level	Country	Safety	Knowledge	Social-Cultural	Economic	Medical access	Others	Research method	Cite
Upper-MICs	Dominica	Safety concerns	Low knowledge, Misinformation	-	Cost barriers	Access issues		Quali	[10]
	Brazil	Safety concerns	Low knowledge	Religious and traditional beliefs	-	Access issues, Lack of vaccine prescription		Quant	[11]
Lower-MICs	Uzbekistan	Negative effects of future fertility	Knowledge gaps, Source reliability	Social and Cultural factors	-	-		Quali	[8]
	Kenya	-	-	Religious, Sexual activity concerns	-	-		Quali	[6]
	Senegal	-	False information	-	-	-	Worker strikes	Quali	[12]
	Cameroon	Safety concerns	Low education, Low knowledge	Religious leaders do not support vaccination	-	-	Distrust in the MOH or pharmaceutical companies	Quant	[13]
	Zimbabwe	Negative effects of future fertility	Low knowledge		-	-		Quant	[14]

Notes: Upper-MICs:upper-middle-income countries; Lower-MICs:lower-middle-income countries; -:Not available; Quali:Qualitative research; Quant: Quantitative research.

Supplementary Table 9. Summary characteristics of equity

Income level	Country	Education	Race-Ethnicity	Age-Gender	Geographic	Other	Research method	Cite
Upper-MICs	Mexico	-	-	-	Highly marginalized areas women		Quali	[2]
Lower-MICs	Tanzania	Girls who are now out-of-school	Ethnic minorities	Girls who are in secondary school	-	HIV + girls	Quali	[15]

Notes:Upper-MICs:upper-middle-income countries; Lower-MICs:lower-middle-income countries; -:Not available; Quali:Qualitative research; Quant: Quantitative research.

Reference

1. Jaspers L, Budiningsih S, Wolterbeek R, Henderson FC, Peters AA. Parental acceptance of human papillomavirus (HPV) vaccination in Indonesia: a cross-sectional study. *Vaccine* 2011;29(44):7785-93.

2. Torres-Poveda KJ, Cuadra-Hernández SM, Castro-Romero JI, Madrid-Marina V. La política focalizada en el programa de vacunación contra el Virus del Papiloma Humano en México: aspectos éticos. *Acta bioethica* 2011;17:85-94.

3. Delany-Moretlwe S, Kelley KF, James S, et al. Human Papillomavirus Vaccine Introduction in South Africa: Implementation Lessons From an Evaluation of the National School-Based Vaccination Campaign. *Global health, science and practice* 2018;6(3):425-38.

4. Yangchen S, Felsher M, Acosta D, et al. Lessons Learned From Bhutan on extending Girls-Only HPV Vaccination Program to Boys: A Qualitative Study. *Asia Pac J Public Health* 2024;36(6-7):580-88.

5. Adeyanju GC, Essoh TA, Sidibe AR, Kyesi F, Aina M. Human Papillomavirus Vaccination Acceleration and Introduction in Sub-Saharan Africa: A Multi-Country Cohort Analysis. *Vaccines (Basel)* 2024;12(5)

6. Vermandere H, Naanyu V, Degomme O, Michielsen K. Implementation of an HPV vaccination program in Eldoret, Kenya: results from a qualitative assessment by key stakeholders. *BMC public health* 2015;15:875.

7. Talabi O, Gilbert H, Fawzi MCS, Anorlu R, Randall T. Examining barriers and facilitators of HPV vaccination in Nigeria, in the context of an innovative delivery model: a mixed-methods study. *BMJ Public Health* 2023;1(1):e000003.

8. Warsi SK, Nielsen SM, Franklin BAK, et al. Formative Research on HPV Vaccine Acceptance among Health Workers, Teachers, Parents, and Social Influencers in Uzbekistan. *Vaccines (Basel)* 2023;11(4)

9. Carlton JG, Marembo J, Manangazira P, et al. Nationwide introduction of HPV vaccine in Zimbabwe 2018-2019: Experiences with multiple cohort vaccination delivery. *PLOS Glob Public Health* 2022;2(4):e0000101.

10. Liebermann E, Devanter NV, Frías Gúzman N, Ompad D, Shirazian T, Healton C. Parent-Level Barriers and Facilitators to HPV Vaccine Implementation in Santo Domingo, Dominican Republic. *J Community Health* 2020;45(5):1061-66.

11. Soares LMC, de Medonça AEO, de Souza DLB, et al. Factors associated with HPV vaccine hesitancy: A nationally representative cross-sectional study. *Vaccine* 2025;59:127278.

12. Casey RM, Adrien N, Badiane O, et al. National introduction of HPV vaccination in Senegal—Successes, challenges, and lessons learned. *Vaccine* 2022;40:A10-A16.

13. Ntonifor MM, Tazinkeng NN, Kemah BL, et al. Factors associated with parental hesitancy towards the human papillomavirus vaccine: a cross-sectional study. *Sci Rep* 2025;15(1):18284.

14. Garon JR, Mukavhi A, Rupfutse M, et al. Multiple cohort HPV vaccination in Zimbabwe: 2018-2019 program feasibility, awareness, and acceptability among health, education, and community stakeholders. *Vaccine* 2022;40 Suppl 1(Suppl 1):A30-A37.

15. Mphuru A, Li AJ, Kyesi F, et al. National introduction of human papillomavirus (HPV) vaccine in Tanzania: Programmatic decision-making and implementation. *Vaccine* 2022;40:A2-A9.
